# Cluster validity indices for automatic clustering: A comprehensive review

**DOI:** 10.1016/j.heliyon.2025.e41953

**Published:** 2025-01-15

**Authors:** Abiodun M. Ikotun, Faustin Habyarimana, Absalom E. Ezugwu

**Affiliations:** a School of Mathematics, Statistics, and Computer Science, University of KwaZulu-Natal, King Edward Avenue, Pietermaritzburg Campus, Pietermaritzburg, 3201, KwaZulu-Natal, South Africa; b Unit for Data Science and Computing, North-West University, 11 Hoffman Street, Potchefstroom, 2520, North-West, South Africa

**Keywords:** Clustering, Cluster validity index, Automatic clustering, Metaheuristic algorithms, Optimization algorithms

## Abstract

The Cluster Validity Index is an integral part of clustering algorithms. It evaluates inter-cluster separation and intra-cluster cohesion of candidate clusters to determine the quality of potential solutions. Several cluster validity indices have been suggested for both classical clustering algorithms and automatic metaheuristic-based clustering algorithms. Different cluster validity indices exhibit different characteristics based on the mathematical models they employ in determining the values for the various cluster attributes. Metaheuristic-based automatic clustering algorithms use cluster validity index as a fitness function in its optimization procedure to evaluate the candidate cluster solution's quality. A systematic review of the cluster validity indices used as fitness functions in metaheuristic-based automatic clustering algorithms is presented in this study. Identifying, reporting, and analysing various cluster validity indices is important in classifying the best CVIs for optimum performance of a metaheuristic-based automatic clustering algorithm. This review also includes an experimental study on the performance of some common cluster validity indices on some synthetic datasets with varied characteristics as well as real-life datasets using the SOSK-means automatic clustering algorithm. This review aims to assist researchers in identifying and selecting the most suitable cluster validity indices (CVIs) for their specific application areas.

## 1 Introduction

Clustering, an unsupervised machine learning technique, is applied to large unlabelled datasets to uncover hidden patterns inherent in the datasets [1]. Clustering algorithms use intra-cluster cohesion and the inter-cluster separation of data objects to partition unlabelled datasets into distinct groups. Cluster validity indices (CVIs) are used to evaluate the quality of the formed clusters.

Cluster validity indices examine the relationship among the attributes of the cluster such as connectedness, cohesion, symmetry, and separation [2]. Different CVIs use different metrics in determining the value of these various cluster attributes. They exhibit different characteristics based on the mathematical model used in determining the value of the various cluster attributes. They are designed to differentiate between inferior and superior clustering [3]. In literature, many cluster validity indices have been reported for evaluating potential clustering solutions of both classical clustering algorithms and metaheuristic-based clustering algorithms.

In data clustering, there are three major criteria for evaluating the potential clustering solutions' quality: external, internal, and relative criteria [4,5]. The internal criteria evaluate the cluster's quality using the dataset's vector qualities such as the data objects' proximity matrix while the external criteria use a pre-specified structure based on the user's intuition which is imposed on the dataset. The basic idea adopted in the relative criteria is based on comparing the resultant cluster structure with other clustering structures obtained using different input parameters within the same algorithm.

According to Ref. [5], the cluster validation approach based on the internal validation criteria is the most used among the three approaches. In validating cluster results using the internal validation criteria, several methods focus on the level of compactness of the object within a cluster and the level of its separateness from other clusters while some other methods called the stability-based validation rely on the clustering algorithm's stability relative to the performance of the different input dataset samples [5].

The dimensionality and density of real-world datasets are known to be high. Therefore, pre-identifying the number of clusters in a dataset is difficult. The automatic clustering approach to data clustering seeks to determine the appropriate cluster number in a dataset with no prior knowledge of the structure of the dataset. It also discovers the corresponding inherent partitioning structure of such a dataset [3]. The automatic clustering problems are expressed as the problem of optimization using optimization techniques to find its solution. Cluster validity indices are usually adopted as fitness functions for evaluating the quality of the potential clustering solutions [3]. Based on some objective function given in a defined domain, optimization finds the best available values that are good enough and best fit for the objective [6].

Metaheuristic optimization is categorized as a higher-level optimization technique that employs simple but efficient methods in finding solutions to optimization problems [7]. Algorithms based on metaheuristic optimization approach have become the latest in finding solutions to optimization problems [6]. The majority of modern optimization techniques involve metaheuristic techniques serving as a powerful tool in providing solutions to hard optimization problems. Their application in major areas of science, engineering, and industrial applications has been well reported in the literature [6].

Cluster validity function is used in metaheuristic-based clustering algorithms as fitness functions The aim is to identify the optimal solutions to the clustering problem based on the data object's intra-cluster cohesion and inter-cluster separation. Different cluster validity indices exhibit varied characteristics that are dependent on different criteria such as the proximity measure, cluster prototype type, and processes involved in measuring the intra-cluster cohesion and inter-cluster separation [3]. Cluster validity indices such as the Xie-Beni, Silhouette, Davies-Bouldin Index, Dunn index, and Calinski-Harabasz index have been used in metaheuristic-based clustering algorithms. In most cases, the choice of CVIs selected for the metaheuristic-based clustering is not based on experimental judgment to support their selection with the behavioural characteristics.

This systematic study is a focused study on the existing cluster validity indices that have been used in metaheuristic-based automatic clustering algorithms as fitness functions. It presents a systematic review of identified CVIs that have been used in metaheuristic-based automatic clustering algorithms reported in the literature. It discusses the strengths and weaknesses of each of the CVIs in their functionality as fitness functions in metaheuristic-based automatic clustering algorithms. The following research questions were addressed in this review.

1. Which of the existing cluster validity indices has been adopted as a fitness function in metaheuristic-based clustering algorithms?
2. Which of the cluster validity indices discovered in RQ1 were mostly used?
3. Are there basic criteria for selecting a cluster validity index for any given metaheuristic-based automatic clustering algorithm?
4. What factors contribute to CVIs evaluation performance?

Does the real-life application area of automatic clustering affect the choice of CVIs? This paper is organized as follows: In section 1, the introduction to the study is presented while Section 2 reports the methodology employed for the systematic study. Section 3 presents the existing related reviews on CVIs in comparison with this current work. The discussion on Automatic clustering and the various cluster validity indices used in metaheuristic-based automatic clustering algorithms is given in Section 4. Section 5 presents the findings from the systematic review as well as the identified application areas. In section 6, the experimental studies and discussions of the findings are presented. The conclusion of the study is presented in Section 7.

## 2 Research methodology

This study aims to conduct a systematic review of the various internal cluster validity indices that have been used as fitness functions in metaheuristic-based automatic clustering algorithms. In this section, the report on the review methodology adopted in the study is presented. For the systematic literature review, the procedure presented by Ref. [8] was adopted. The details of the selection processes concerning the database search, the search keywords, search techniques, and data sources as well as the inclusion and exclusion criteria for the identification of relevant research papers are presented to buttress the transparency of the selection process.

### 2.1 Search keywords

To retrieve the most relevant research papers that assist in providing answers to our research questions, keywords that are common to the research purpose were used in the search process. The list of keywords used includes cluster validity indices, automatic data clustering, metaheuristic optimization algorithm, cluster separability measure, cluster evaluation criteria, clustering performance analysis, and cluster validity concepts. The names of the various identified cluster validity indices were also used to find relevant literature that reports on their use in any metaheuristic algorithms for automatic clustering. These keywords were used to search the relevant academic databases for the articles included in the review.

### 2.2 Article search

**Fig. 1:**
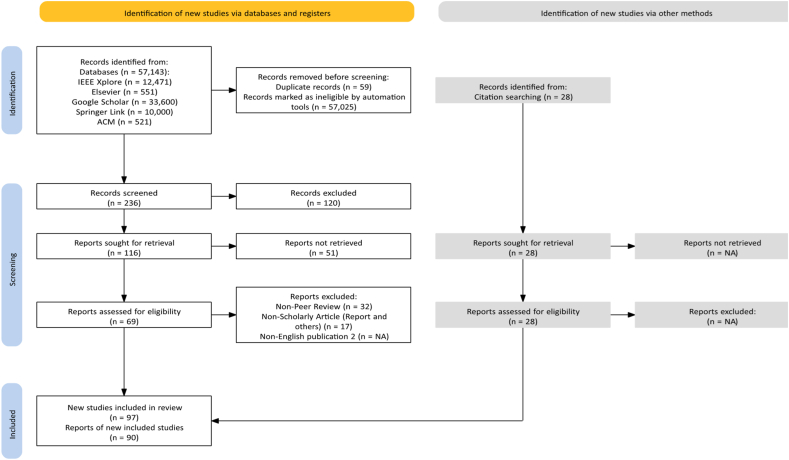
Literature search and selection process prisma diagram.

The search for the relevant articles was carried out between February 2024 and May 2024. A total number of 57143 articles were identified during the initial automated search from the various databases. 57081 articles were filtered out using the electronic database advanced search combining the various keywords with the ‘OR’ and ‘AND’ options to further streamline the retrieved articles leaving 90 articles for the review. The citations and references of the retrieved articles were further scanned for more related articles with 28 articles added. The PRISMA [9] diagram reflecting the search and selection process is presented in Fig. 1.

### 2.3 Academic databases

In searching for the relevant articles, the search focused on credible sources including conference proceedings, peer-reviewed journals, and edited books that were indexed in various academic databases. The academic databases used for the extraction of the relevant articles include Springer, IEEE Xplore, Google Scholar, Elsevier, and ACM Digital Library. These repositories keep high-quality, SCI-indexed journal publications and top international conferences.

### 2.4 Article inclusion/exclusion criteria

Each article was evaluated based on the title, abstract, full content, and conclusion to verify if it aligns directly with the review objectives and goals. The details of the inclusion and exclusion criteria presented in Table 1 were used to ensure that the most relevant articles were included in the selection.

**Table 1:** Systematic review selection criteria.

Inclusion	Exclusion
The article focused on Metaheuristic-based Automatic clustering to ensure that only articles that aligned with the research objectives and goal were selected.	Articles on classical clustering and other clustering approaches were not considered.
Articles that used internal cluster validity indices for automatic clustering	Articles that use other mode of validity indices i.e. external and relative were excluded.
Conference proceedings, peer-reviewed journals, and edited books published in reputable journals were included to ensure the use of academic-level sources and the quality of relevant literature.	Non-peer-reviewed articles, reports, and other sources were excluded.
Articles published in the English language only were included to keep with the official language of research articles and to ensure a proper understanding of the article content.	Articles published using any other language apart from English are excluded.

## 3 Comparison with existing survey on cluster validity indices

The main differences between existing surveys on cluster validity indices and this systematic review work are presented in this section. Several literatures have been published on different cluster validity indices with many introducing new cluster validity indexes or improving the existing ones. Comparative analysis of some of these cluster validity indices has been reported with a view of evaluating their performances to specific clustering algorithm categories (classical clustering algorithms or nature-inspired metaheuristics) and their performances based on the characteristic nature of the datasets. For instance Ref. [10], published a survey of Fuzzy clustering validity evaluation methods. The authors in Refs. [3,[11], [12], [13], [14], [15]] present a comparative analysis of CVIs based on the classical clustering algorithms. Publications reporting comparisons of cluster validity indices include [[16], [17], [18], [19], [20], [21]].

For automatic clustering algorithms, there are reviews and survey studies reported in the literature that discussed some of the clustering similarity measures used in metaheuristics-based automatic clustering [22,23]. The authors in Ref. [24] mentioned 17 validity indices that have been used as fitness functions in metaheuristic-based automatic clustering. The work of [25] mentioned 25 different internal cluster validation measures and eight external cluster validation measures. The performances of 68 cluster validity indices were reviewed by Ref. [26] on 21 real-life and simulated datasets. Their evaluation was based on multivariate chemometric methods for disclosing the mutual relationship among the indices and reporting their effectiveness in terms of accuracy and reliability. Their discussion was based purely on the general performance of the CVIs and not particularly about automatic clustering. They intended to present a survey of most of the CVIs used for crisp clustering comparing their performances from a multivariate chemometric perspective. Table 2 presents a summary of the existing survey and comparative analysis of cluster validity indices comparing them with this systematic study.

**Table 2:** Summary of existing cluster validity indices surveys.

Author and year	Publication year	Study focus	Impact as of 2024
[27]	1985	The study used an agglomerative process of hierarchical clustering for comparative analysis of CVIs	5290
[28]	1987	The study focused on a comparative study of two internal indices in estimating the true number of clusters in multivariate data to show their effectiveness.	371
[29]	1997	Comparative studies of CVIs for the choice of the correct number of components in a mixture of normal distributions.	126
[14]	2002	Comparative study similar to Milligan & Cooper's work with a focus on choosing the correct number of components using cluster validity indices based on high dimensional empirical binary data	416
[12]	2007	Examined CVIs' correlation with the error rates	310
[11]	2011	Comparison of CVIs using a different methodology that avoids false assumptions based on the correctness of the clustering algorithms	93
[5]	2013	Extensive comparative study of the performance of 30 CVIs	1414
[30]	2021	Compared external and internal cluster validity indices with a similar bounded index range.	3
[15]	2012	Comparison of CVIs using Swarm intelligence-based clustering.	153
[24]	2021	Survey of CVIs for automatic data clustering using ACDE.	14
[31]	2021	Study popular CVIs to determine their suitability or unsuitability for judging the quality of different partitions of the same cardinality.	26
[26]	2024	Compared 68 cluster validity indices using the K-means clustering algorithm using multivariate chemometric methods	1
This work	2024	The study focused on internal validity indices used as fitness functions in metaheuristic-based automatic clustering algorithms using SOSK-means	

## 4 Metaheuristic-based automatic clustering algorithm and clustering validity indices

The problem of automatic clustering heralded a new era in cluster analysis in the late 1990s because of the proliferation of big data which are mostly unlabelled. The automatic clustering algorithms find the optimal number of clusters in a dataset automatically while at the same time grouping the data objects into appropriate clusters [2]. Metaheuristic search algorithms were identified as the techniques mostly used for automatic clustering algorithm implementation [24]. In metaheuristic-based automatic clustering, the clustering problems are treated as optimization problems to minimize the intra-cluster distance and maximize the inter-cluster distances [32].

Several successful implementations of metaheuristic algorithms for automatic clustering problems have been widely reported in the literature [[33], [34], [35], [36], [37], [38], [39], [40], [41]]. A survey on the use of nature-inspired metaheuristic algorithms in finding solutions to automatic clustering problems was conducted by the authors in Ref. [33]. The authors in Ref. [42] classified metaheuristics-based clustering algorithms as search-based, hard partitional clustering algorithms and were subdivided into evolutionary-based e.g. Genetic algorithms, swarm intelligence e.g. Particle swarm Optimization, and others.

There are two major problems associated with solving automatic clustering problems: finding the optimal cluster numbers and all data groups' correct identification. The clustering task is known to be computationally expensive even for moderately sized problems [32,33,43]. The problem of finding an optimal clustering solution when K>3 is an NP-hard problem. Given N objects with K clusters, N objects partitioned into K clusters will require the following number of combinations as represented in Equation (1).

(1) $$C\left(N,K\right)=\frac{1}{K!}\sum_{i=0}^{K}{\left(-1\right)}^{K-i}\left(\frac{K}{i}\right)i^{N}$$

and to find the optimal number of clusters, the search space is given Equation (2).

(2) $$O\left(N\right)=\sum_{K=1}^{N}S\left(N,K\right)$$

Automatic clustering seeks to find an optimal number of clusters within a defined range $\left[K_{\min},K_{\max}\right]\text{.}$ The automatic clustering problem based on metaheuristic optimization technique is formulated as an optimization problem given in Equation (3):

Given

(3) $$\Omega =\left\{{∁}^{1},{∁}^{2},\cdots {∁}^{B\left(n\right)}\right\}$$

as the set of all clustering that is possible where the clustering solutions represent each element of the set for a given dataset N with f given as the single fitness function (the cluster validity index serves as the fitness criterion). For a single objective clustering problem, (Ω,f) is required to find the clustering solution ${∁}^{*}$ as defined in Equation (4), where:

(4) $$f\left({∁}^{ˆ}*\;\right)=\min\;\{f\left(∁\right)\;|∁\in \Omega \}$$

Such that f(C) is minimized without loss of generality. For a multi-objective clustering problem, $\left(\Omega ,f_{1},{\;f}_{2},{\cdots ,f}_{m}\right)$ is required to find the clustering solution ${∁}^{*}$ that satisfies Equation (5).

(5) $$f\left({∁}^{ˆ}*\;\right)=\min\;\left\{f_{t}\;\left(∁\right)\;|∁\in \Omega \right\},t=1,2,\cdots ,m$$

where $f_{t},t=1,2,\cdots ,m$ represents the set of m (single) criterion functions. The multi-objective problems usually return multiple optimal solutions for which the principle of Pareto dominance is used in identifying the solutions. The principle of Pareto states that given ${∁}_{1},{∁}_{2}\in \Omega ,{∁}_{1}$ is regarded as a dominating ${∁}_{2}$ if and only if Equations (6), (7) holds.

(6) $$f_{t}\left({∁}_{1}\right)\leq f_{t}\left({∁}_{2}\right),\forall t\in 1,2,\cdots m$$

and

(7) $$f_{t}\left({∁}_{1}\right)\;<\;f_{t}\left({∁}_{2}\right),\forall t\in 1,2,\cdots m$$

All Pareto nondominated solutions form the Pareto-optimal set and the objective function values corresponding to this set are called the Pareto-optimal front.

### 4.1 Cluster validity methods for automatic clustering algorithms

The quality of potential clustering solutions is evaluated using cluster validity index and it also determines the optimal cluster numbers in automatic clustering problems. Specifically, the quality of clusters is typically determined using internal cluster validity measures, external validation methods, and domain-specific evaluation techniques, each of which is explained in detail subsequently. It is noteworthy that while CVIs like the Davies Bouldin Index, Compact-Separated index, Silhouette index, or Dunn index help assess the validity of the number of clusters, they also provide insight into cluster quality by evaluating metrics such as compactness and separation, both of which has been extensively employed in the literature to determine the quality of clustering task. While the compactness metrics, measure how closely related or tight the points are within a cluster (e.g., based on intra-cluster distances), the separation metrics measure how distinct clusters are from each other (e.g., based on inter-cluster distances). Further explanations are provided subsequently in the next paragraphs.

Several cluster validity indices have been proposed in literature with new ones being introduced as better alternatives to existing ones. According to Ref. [10], CVI research has become a hot topic. The clustering validity indices are broadly grouped into three categories: the external validity methods, the internal validity methods, and the relative validity methods [10,23,25]. The external validity uses a pre-specified structure based on the user's intuition which is imposed on the dataset. The clustering results are compared with previously known structures obtained using similar parameters based on some external information such as the class labels.

The internal criteria evaluate clusters' quality using the dataset's vector qualities such as the proximity matrix of the data objects. The underlying structure of real-life datasets is usually not known and as such it is difficult to know the correct number of clusters that will be optimal for the dataset. The internal cluster validity methods are mostly used in metaheuristic-based automatic clustering algorithms to estimate the correct number of clusters in each dataset. They do not depend on any prior clustering structure of the dataset. They evaluate clustering results using some defined formulas which are based on various factors such as dataset density, skewed distribution, noise, sub-clusters, and monotonicity of index.

The internal cluster validity methods measure the intra-cluster compactness and the inter-cluster separation. The intra-cluster compactness determines the homogeneity of a single cluster, and the similarity level of data objects within the same cluster, while the inter-cluster separation measures the heterogeneity of the different clusters, measuring how different the data objects in different clusters are to each other. Cluster compactness is commonly measured using intra-cluster distance, within-group dispersion, or variance which are usually required to be minimized [26]. The inter-cluster separation measures how far apart clusters are, and the metrics used for this include the use of the nearest neighbour distance, the farthest neighbour distance, and the distance between the clusters’ centroids. According to Ref. [44], inter-cluster separation plays a more important role in cluster validation than intra-cluster cohesion.

The internal validity indices that have been used in metaheuristic-based automatic clustering algorithms are discussed below. The summary of the identified cluster validity indices is presented in Table 3.

**Table 3:** Summary of Cluster Validity Indices that have been applied to Metaheuristic-based automatic clustering algorithms.

SN	Cluster Validity Indices	Optimum index value rule	Strength	Weakness
1	Baker-Hubert Gamma index [45]	Maximum difference	The Baker-Hubert Gamma index is sensitive to the true underlying clustering structure. It effectively distinguishes between random and meaningful clustering by offering a robust measure of how well the clustering algorithm has captured the inherent pattern in the datasets.	computationally prohibitive and impractical for most real applications of cluster analysis
2	Ball-Hall index [46]	Maximum difference	No absolute threshold is used in the measure of similarity criterion of this technique. The technique is independent of the sequence in which patterns are presented [100]. Capable of finding correct clustering structure for arbitrarily shaped clusters with high density [101,102]	The use of metrics weighted with respect to cluster as well as component can make clustering interpretation difficult when used for data analysis
3	Bandeld-Raftery index	Maximum difference	The index incorporates a penalty for the number of parameters in the model, helping the model to prevent overfitting. Also, by using the likelihood function, the index evaluates how the model fits the data. Lastly, the model can be used with large datasets and complex models.	Calculating the index can be extremely compute-intensive, especially for large datasets. Moreover, the effectiveness of the index relies on the correctness of the underlying models.
4	Bayesian Information Criterion Index [48]	Minimization	BIC supplies computationally inexpensive proxies to otherwise difficult-to-calculate posterior model probabilities [103].	This technique has a strong distribution assumption of parametric likelihood [104]
5	C-Criterion Index [48]	Minimum	C-Criterion primarily measures the model prediction accuracy with a statistical significance of optimal unbiased estimator of linear combinations of parameters [105]	The calculation of the C criterion does not yield a specific value but instead ranks designs by comparing their C criterion vectors [105].
6	Calinski-Harabasz Index [52]	First maximum	It uses the arrangement of clusters to assess the quality of the clustering solution regardless of the choice of distance measure.	The Calinski–Harabasz index is shown to be affected by the data size and level of data overlap. It is regarded as data dependent such that its behaviour may change if different data structures are used for the same datasets [27]. Only applicable to spherical clusters [106]
7	Category Utility Metric [57]		There is a reduction of uncertainty due to the communication of category information through some cues [107].	There is the assumption that probability distributions on separate attributes are statistically independent of one another which is, however, not always true because the correlation between attributes often exists [108].
8	Compact-Separated index [62]	Minimization technique	Efficient in handling clusters with different dimensions, densities, or sizes Produces more good quality solutions	Computationally intensive and expensive
9	Condorcet's Criterion [65]	Maximization Technique	It uses a natural cluster structure without the need to use sampling methods of data that can lead to inaccurate results [109].	It involves handling large matrices of o(n^2^) complexity. There is a need to fix some initial parameters such as the number of iterations and the similarity threshold [109].
10	COP index [5]	Minimum	The COP is not affected by the number of clusters and is hardly affected by cluster overlap [5]	Only applicable to spherical clusters [106]
11	Davies-Bouldin Index [50]	Minimization technique	Hardly affected by cluster overlap [5]. Demonstrates a good clustering partition.	Make strong assumptions that are not valid in many real situations [110]. Too simple to handle data with specific structures such as arbitrarily shaped with dispersed density. Only applicable to spherical clusters [106]
12	S_Dbw validity index [21]	First Minimum	Work well for compact and well-separated clusters. Robust to noise [5].	Can not work with non-convex clusters or clusters with extraordinary, curved geometries. High computational Cost [93]
13	Det Ratio index [76]	Minimum difference	One of the best validity criteria for arbitrarily shaped closed contour clusters [102]. Capable of finding correct clustering structure for arbitrarily shaped clusters with high density [101]	Det Ratio index can be highly sensitive to the size and shape of the clusters. More so, it does not explicitly account for the overlap between clusters.
14	Dunn index [72]	Maximum	Capable of finding correct clustering structure for arbitrarily shaped clusters with high density [101,102]	Make strong assumptions that are not valid in many real situations [110]. Difficulty with handling arbitrarily shaped clusters and clusters with dispersed density due to their general simplicity Computationally expensive and sensitive to noise. Only applicable to spherical clusters [106]
15	Gamma index [45]	Maximum	Suitable for datasets with compactness properties and datasets with multiple densities [111]	Data-dependent varied behaviour per data structure [27] Computationally expensive. Inefficient with overlapping clusters. Difficulties with arbitrarily shaped clusters [97]
16	Generalized Dunn index [44]	Maximum	Good for validating hyper-spherical/cloud and shell-type clusters [44].	Computationally intensive and expensive [68,112]
17	G-plus index [75]	Minimum	Capable of finding correct clustering structure arbitrarily shaped clusters with high density [101]	Computationally expensive. Inefficient with overlapping clusters. Difficulties with arbitrarily shaped clusters [97,112]
18	I-index	Maximum	I is found to be more consistent and reliable in indicating the correct number of clusters compared with DB, CH, and DI [113]	Requires parameter tunning [114]
19	Ksq_DetW index [76]	Maximum difference	Capable of finding correct clustering structure for arbitrarily shaped clusters with high density [101]	Does not allow for direct comparison between clustering algorithms [115]
20	Log_Det_Ratio index [76]	Minimum difference	Capable of finding correct clustering structure arbitrarily shaped clusters with high density [101,102]	The Log_Det_Ratio index assumes that clusters are roughly spherical and of similar size. It also focuses more on the compactness of clusters, potentially neglecting other essential aspects of clustering quality such as separation between clusters.
21	Log_SS_Ratio index [78]	Minimum difference	Capable of finding correct clustering structure arbitrarily shaped clusters with high density [101,102]	Outliers can significantly affect the within-cluster sum of squares, distorting the measure of cluster compactness.
22	McClain-Rao index [77]	Maximum difference	Perform relatively well in low dimensions [116].	Performance degrades as the dimension increases [116]. Worst performing CVI [11]
23	Negentropy Increment [80]	First Minimum	Calculation Simplicity. Satisfactory performance on clusters with heterogeneous orientation, densities, and scales. Assess the correct number of clusters with more reliability than DB, Dunn, and PBM [117]	Poor performance with datasets with low number of data points [118].
24	Niva index [82]	Minimum	Takes advantage of cluster density, size, and shape [82].	The index can often place too much emphasis on certain metrics, such as within-cluster variance, potentially neglecting other important aspects of clustering quality, such as the overall structure or topology of the data.
25	OS-index [83]	Minimum	Efficient for clusters of different shapes, sizes, and density	Poor performance with overlapping clusters
26	PBM index [17]	Maximum	It favours more compact and fewer clusters.	Only capable of identifying compact clusters
27	Point-Biserial Index [85]	Maximum	Capable of finding correct clustering structure for arbitrarily shaped clusters with high density [101]	Sensitivity to varying numbers of clusters or dimensions in datasets [68]
28	Ratkowsky-Lance index [86]	Maximum	Superior performance in validating clusters in binary datasets [14]	Weakness in correct absolute cluster profile identification [14]
29	Ray-Turi index [87]	Minimum	Demonstrate Superior performance in cluster validation for dynamic connectivity data [119]	Exhibit Sensitivity problem [120]
30	Root-mean square standard deviation [121]	Minimum	Valid for rectangular data [122]	Only valid if the method used is average, centroid, and ward [122] Can only validate well separated hyper sphere-shaped clusters [123]
31	Scatter Criterial [89]		The Scatter Criterion is relatively simple to understand and compute. It can also be applied to a variety of clustering algorithms, making it versatile in its use.	The criterion primarily focuses on within-cluster compactness and does not explicitly consider the separation between clusters.
32	Score function [90]	Maximum	Good for validating hyper-spheroidal clusters as well as multidimensional and noisy datasets. It can handle single cluster case and sub-cluster hierarchies [114]	Restricted to datasets containing hyper-spheroidal clusters
33	Scott-Symons index [76]	Minimum	Suitable for clusters of different shapes, sizes, and orientations [26],	Where clusters are not well represented, it cannot be properly calculated [26]. Not robust to noise [47]
34	SD validity index [4]	Minimization	Find Optimal Partition independent of the clustering algorithm [70]	Sensitive to the geometry of the cluster centres and number of clusters [26]
35	Silhouette Index [94]	Maximization	Depends only on the actual partition of objects and not on the clustering algorithm. Useful for improving cluster analysis results. For comparison of clustering solution of different clustering algorithms. Suitable for datasets with compactness properties and datasets with multiple densities [111]	It is related to specific distance measures and so cannot be used for comparing with clustering results that use different distance measures. Only applicable to spherical clusters [106]
36	Sum of Squared Error [96]	Maximum rate of change	It provides a clear numerical value that indicates the compactness of clusters. It can be used with various clustering algorithms, such as k-means, hierarchical clustering, and others. It is a versatile measure that can be applied across different methods.	The index is highly sensitive to outliers, as they can significantly increase the total error. Calculating the index for very large datasets or high-dimensional data can be computationally expensive. This can limit its practicality for large-scale clustering tasks.
37	SV-Index [97]	Maximization technique	Independent of the number of objects in a cluster, data density, is less dependent on cluster centroid and average values. Efficient handling of clusters of different sizes and densities [97]	Calculating the SV-Index can be computationally intensive, as it often requires multiple runs of the clustering algorithm and comparisons between results. This can be time-consuming, especially for large datasets or complex algorithms.
38	Sym-index [18]	Maximization technique	Efficient at detecting symmetrically shaped clusters [18]	Dependent on the underlying clustering algorithm [18]. Only applicable to internally symmetric datasets [106]
39	Tau index [92]	Maximization technique	Capable of finding correct clustering structure arbitrarily shaped clusters with high density [101]	High computational cost [112]
40	Trace_W index [92]	Maximization technique	Capable of finding correct clustering structure arbitrarily shaped clusters with high density [101]	The index itself may not always provide intuitive insights into the clustering quality, making it challenging to understand the underlying reasons behind its score.
41	Trace_WiB index [98]	Maximization technique	The Trace_WiB index is normalized, which helps in comparing clustering results across different datasets or clustering methods, providing a more standardized measure of cluster validity.	The index might be influenced by the initial conditions, or the clustering algorithm used, leading to variability in results if different algorithms or initializations are applied.
42	Wemmert-Gancarski index	Maximization technique	Performance stability in all distance measures for syntactic and real datasets	Performance sensitivity to noise
43	Xie-Beni index [99]	Minimization technique	Effective detection of hyper-spherical shaped clusters [18]	Decreases monotonically when the number of clusters is very large [97]

**Baker-Hubert Gamma index**: Baker-Huberts Gamma index [45] evaluates the correlation between two vectors X and Y whose dataset size is the same. The Γ index is adapted in the Baker-Hubert Gamma index and the definition is given as in Equation (8):

(8) $$C=\Gamma =\frac{S^{\pm S^{-}}}{S^{+}+S^{-}}$$

where $S^{-}=\sum_{\left(r,s\right)\epsilon I_{Y}}\sum_{\left(u,v\right)\epsilon I_{Y}}1_{\left\{d_{uv}>d_{rs}\right\}}$ and $S^{+}=\sum_{\left(r,s\right)\epsilon I_{Y}}\sum_{\left(u,v\right)\epsilon I_{X}}1_{\left\{d_{uv}<d_{rs}\right\}}$

The Baker-Huber Gamma index has a computational complexity of $O\left(n^{2}logn\right)$. The pairwise distance calculation between the two vectors makes it computationally intensive and unsuitable for large datasets.

**Ball-Hall index**: The Ball-Hall index [46] measures the mean of the mean dispersion of all the clusters. It is given as shown in Equation (9):

(9) $$Ball-Hall\;index\left(C\right)=\frac{1}{K}\sum_{K=1}^{K}\frac{1}{n_{k}}\sum_{i\epsilon I_{k}}{\left\|{M_{i}}^{\left\{k\right\}}-G^{\left\{k\right\}}\right\|}^{2}$$

It is the average counterpart of the Trace_W index [26]. The computational complexity of the Ball-Hall index is O(n.d) which accounts for the centroid calculation and the variance calculation. The linear complexity makes it relatively efficient for large datasets.

**Banfield-Raftery index:** The Banfield-Raftery index [47] uses the variance-covariance of each cluster to measure the performance of the clustering result. In the Banfield-Raftery index, the logarithms' weighted sum of the variance trace of each cluster's covariance matrix is measured and it is defined in Equation (10):

(10) $$C=\sum_{k=1}^{K}n_{k}\;\log\;\left(\frac{Tr{\left(WG\right)}^{\left\{k\right\}}}{n_{k}}\right)$$

Banfield-Raftery index is proposed as an alternative index to the Trace_W index using the square of the average distance from the centroids of the clusters instead of the sum of squares criterion used in the Trace_W index. It produces a better performance by finding varied sizes of hyper-spherical clusters. The cluster size is measured using the volume occupied and not the number of objects within the cluster. It has a computational complexity of $O\left(n.d^{2}+k.d^{3}\right)$ which makes it computationally expensive for high dimensional datasets and datasets with large numbers of clusters.

**Bayesian information criterion (BIC) index:** The Bayesian information criterion (BIC) [48] index is a minimization problem that tries to solve partitions’ overfitting problems of the clustering algorithm. The definition of BIC is given as shown in Equation (11):

(11) BIC=−ln(L)+vln(n)

where L represents the likelihood of data generation by the parameters in the model, n represents the number of entities and v represents the number of free parameters in the Gaussian model. The computational complexity of the Bayesian information criterion is $O\left(n.d+k.d^{2}\right)$. It is efficient for datasets with a moderate number of dimensions and the number of clusters.

**C-criterion:** The C-criterion [49] is an extension of Condorcet's validity index. It compares the maximum and minimum possible intra-cluster distances with the total intra-cluster distances for a given dataset. The definition is given as represented in Equation (12):

(12) $$\sum_{C_{i}\epsilon C}\sum_{\begin{array}{c} x_{j},x_{k}\epsilon C_{i}\\ x_{j}\neq x_{k} \end{array}}\left(s\left(x_{j},x_{k}\right)-\gamma \right)+\sum_{C_{i}\epsilon C}\sum_{\begin{array}{c} x_{j}\epsilon C_{i};\\ x_{k}\notin C_{i} \end{array}}\left(\gamma -s\left(x_{j},x_{k}\right)\right)$$

The computational complexity is $O\left(n^{2}\;\log\;n\right)$ [50,51]**.**

**Calinski-Harabasz index:** In the Calinski-Harabasz index [52], the cluster's closeness or compactness is measured based on the distance between the cluster's centroid and the data points within the cluster while the cluster's separation from other clusters is measured using the distance from the cluster's centroid to the global centroid [2]. The definition of the Calinski-Harabasz validity index is given as Equation (13):

(13) $$CH=\frac{trace\left(S_{B}\right)}{trace\left(S_{w}\right)}*\frac{n_{p}-1}{n_{p}-k}$$

where $\left(S_{w}\right)$ is the intra-cluster scatter matrix, $\left(S_{B}\right)$ is the inter-cluster scatter matrix, k is the number of clusters and $n_{p}$ is the number of data objects in a cluster. It is known to be data-dependent such that its behaviour may change if different data structures are used for the same datasets [27]. The CH index has a linear computational complexity O(n.d) which makes it very efficient for large and high dimensional datasets [51,52]. The variants of the CH index include the LSSR index [53], the Ratkowsky-Lance (RL)index [54], the RS index [55], and the WCH index [56]. The LSSR is a logarithmic scale-based variant that measures the logarithmic ratio of the sum of the inter-cluster squared distance to the sum of the squared intra-cluster distance. The RL index variant considered the mean value of the ratios obtained for each dataset object. The RS index variant finds the extent to which the differences between clusters differ from each other. In the WCH index variant, consideration is given to large overlaps among clusters using a correction factor that accounts for these overlaps among the clusters. The CH index uses cluster arrangement to assess the quality of the clustering solution regardless of the choice of distance measure.

**Category utility metric** [57]: The measure of the goodness of a category is evaluated by the Category utility metric. Given a set of entities, the binary category $C=\left\{c,\bar{c}\right\}$ is defined in Equation (14).

(14) $$CU\left(C,F\right)=\left[p\left(c\right)\sum_{i=1}^{n}p\left(f_{i}|c\right)\log\;p\left(f_{i}|c\right)+p\bar{\left(c\right)}\sum_{i=1}^{n}p\left(f_{i}|\bar{c}\right)\log\;p\left(f_{i}|\bar{c}\right)\;\right]-\sum_{i=1}^{n}p\left(f_{i}\right)\log\;p\left(f_{i}\right))$$

where n-sized binary feature set is given in Equation (15):

(15) $$F=\left\{f_{i}\right\},i=1,2,\ldots \text{,}$$

and p(c) represents an entity prior probability of belonging to the positive category c;

$p\left(f_{i}|c\right)$ represents the conditional probability that the feature $f_{i}$ belong to the positive category c;

$p\left(f_{i}|\bar{c}\right)$ represents the conditional probability that the feature $f_{i}$ belong to the positive category $\bar{c}\text{;}$

$p\left(f_{i}\right)$ represents the entity's previous probability (Corter and Gluck, 1992; Ezugwu et al., 2020a).

The Category utility metric has a linear computational complexity and it is given as O(n.d+k.d.m) with the m representing the average number of possible values per attribute [51,58].

**C-index:** The definition of the C-index cluster validation method [59] is given in Equations (16), (17), (18), (19).

(16) $$CI\left(C\right)=\frac{S\left(C\right)-S_{\min\left(C\right)}}{S_{\max\left(C\right)}-S_{\min\left(C\right)}}$$

where:

(17) $$S\left(C\right)=\sum_{C_{k}\in C}\sum_{x_{i},x_{j}\in C_{k}}d_{e}\left(x_{i}x_{j}\right)$$

and

(18) $$S_{\min\left(C\right)}=\sum \min\;{\left(n_{w}\right)}_{x_{i},x_{j}\in X}\left\{d_{e}\left(x_{i}x_{j}\right)\right\}$$

and

(19) $$S_{\max\left(C\right)}=\sum \max\;{\left(n_{w}\right)}_{x_{i},x_{j}\in X}\left\{d_{e}\left(x_{i}x_{j}\right)\right\}$$

The overall computational complexity of the C-index is $O\left(n^{2}\;\log\;n\right)$ [51,60,61].

**Compact-Separated (CS) index:** The Compact-Separated (CS) index [62] gives the ratio of the sum of within-cluster scatter to between-cluster separation. Suppose the distance measure V is given as $V\left(X_{i},X_{j}\right)$ and the intra-cluster scatter is given as $X_{i}$ with the inter-cluster separation is given as $X_{j}$, the CS index for clustering Q is calculated as described in Equations (20), (21).

(20) $$CS\;\left(Q,V\right)=\frac{\frac{1}{P}\sum_{i=1}^{P}\left[\frac{1}{D_{n}}\sum_{X_{i\in Q_{i}}}\;\max_{X_{j\in Q_{i}}}\left\{V\left(X_{i},X_{j}\right)\right\}\right]}{\frac{1}{P}\sum_{i=1}^{P}\left[\;\min_{j\in P,j\neq i}\;\left\{V\left(x_{i},x_{j}\right)\right\}\right]}$$

(21) $$=\frac{\sum_{i=1}^{P}\left[\frac{1}{Q_{i}}\sum_{X_{i\in Q_{i}}}\;\max_{X_{j\in Q}}\left\{V\left(X_{i},X_{j}\right)\right\}\right]}{\frac{1}{P}\sum_{i=1}^{P}\left[\;\min_{j\in P,j\neq i}\;\left\{V\left(x_{i},x_{j}\right)\right\}\right]}$$

where:

$V\left(X_{i},X_{j}\right)$ represents the distance between the within-cluster scatter $X_{i}$ and the between-cluster separation $X_{j};P$ represents the number of clusters in Q and the number of data points in cluster P is given as $\left|D_{n}\right|$ with the distance of data points from their centroids given as d. The computational complexity for the CS index is given as $O\left(n\;.\;d+k^{2}\;.\;d\right)$ [51,61,63]. According to Ref. [64], the CS index is reported as being more efficient in handling clusters with different densities or sizes and dimensions. It produces good quality solutions when compared with the DB index. In terms of execution time, however, it is more computationally intensive. The CS index has the same computational complexity as the K-means when the number of clusters is far smaller compared with the total number of data objects in the dataset.

**Condorcet's criterion:** Condorcet's criterion [49,65] is defined as given in Equation (22).

(22) $$\sum_{C_{i}\in C}\sum_{\begin{array}{c} x_{j},x_{k}\in C_{i}\\ x_{j}\neq x_{k} \end{array}}s\left(x_{j},x_{k}\right)+\sum_{C_{i}\in C}\sum_{x_{j}\in C_{i};x_{k}\notin C_{i}}d\left(x_{j},x_{k}\right)$$

Condorcet's criterion has a computational complexity $O\left(n\;.\;m^{2}\right)$ where m is the number of candidates [66,67].

**COP Index:** The Clustering Outcome Prediction (COP) index is a measure of the distance between the cluster points and the centroid and the largest distance between neighbours gives the separation measure [5]. The definition is given in Equation (23).

(23) $$COP\left(C\right)=\frac{1}{N}\sum_{x_{i}\in c_{k}}\left|c_{k}\right|\frac{\frac{1}{\left|c_{k}\right|\sum_{x_{i}\in c_{k}}d_{e}\left(x_{i}\bar{c_{k}}\right)}}{\min_{x_{i}\notin c_{k}}\;\max_{x_{i}\in c_{k}}d_{e}\left(x_{i}x_{j}\right)}$$

It has an overall computational complexity of $O\left(n\;.\;d+k^{2}\;.\;d\right)$. In datasets with k≪n, the complexity is approximately linear with respect to the number of data points [51,68].

**Davies-Bouldin Index (DB):** Davies-Bouldin Index [50] finds the mean inter-cluster similarity between any two clusters and their nearest. DB is minimized for a better result. The DB index is defined in Equation (24).

(24) $$BD=\frac{1}{c}\sum_{i=1}^{c}\max_{i\neq j}\left\{\frac{d\left(x_{i}\right)+d\left(x_{j}\right)}{d\left(c_{i},c_{j}\right)}\right\}$$

where i and j represent cluster labels, $d\left(x_{i}\right)$ and $d\left(x_{j}\right)$ represents entities in respective clusters, c represents the number of clusters, and $d\left(c_{i},c_{j}\right)$ represents the distance between cluster centroids. From the study reported by Ref. [69], the DB index is said to be more reliable when the variance on the dataset is equal to 0.16. This indicates that it works better on compact clusters. The DB index has a computational complexity of $O\left(n\;.\;d+k^{2}\;.\;d\right)$ similar to the COP index and this makes the complexity roughly linear for most practical application where k≪n [50,51]. Variants of DBI include the DB2 which measures the mean of the sum of all the clusters of the largest sum ratio of the two clusters radii to the smallest distance between their centroids.

**S_Dbw validity index:** The underlying characteristics of the clusters are used by the S_Dbw validity index [21] to validate the clustering algorithm result. The cluster's compactness is measured using the intra-cluster variance while the clusters' separation is determined based on the inter-cluster density. The definition is given as given in Equations (25), (26).

(25) $$S_{Dbw\left(n_{c}\right)}=Scat\left(n_{c}\right)+{Dens}_{bw\left(n_{c}\right)}$$

where

(26) $${Dens}_{bw\left(n_{c}\right)}=\frac{1}{n_{c}.\left(n_{c}-1\right)}\sum_{i=1}^{n_{n}}\left(\sum_{\begin{array}{c} j=1\\ i\neq j \end{array}}^{n_{c}}\frac{density\left(u_{ij}\right)}{\max\left\{density\left(v_{i}\right),density\left(v_{j}\right)\right\}}\right)$$

where $c_{i},c_{j}$ are clusters with centroids $v_{i},v_{j}$ respectively and the middle point of the line segment is represented by $u_{ij}$. The computational complexity of the S_Dbw index is given as $O\left(n\;.\;d+k^{2}\;.\;d\right)$ similar to the COP and the DB index. It exhibits a linear computational complexity with respect to the number of data objects in the datasets and demonstrates a quadratic computational complexity with respect to the number of clusters [16,51,70].

**Det Ratio index:** Det Ratio index (Scott and Symons, 1971) is given as represented in Equation (27).

(27) $$\mathrm{Det}\;Ratio=\frac{\det\;\left(T\right)}{\det\;\left(WG\right)}$$

Where WG is the individual matrices and T represents the total scatter matrix. The Det Ratio index has a computational complexity of $O\left(n\;.\;d^{2}+d^{3}\right)$ [27,71].

**Dunn index:** Dunn index [72] measures the smallest between-cluster distance and the largest within-cluster distance ratio in a partition. It is a maximization problem and the time complexity is high with respect to the number of data points in the datasets. The computational complexity is given as $O\left(\;n^{2}\right)\text{,}$ [16,51,73]. It is also affected by noise. Dunn index is given as shown in Equation (28).

(28) $$Dunn=\min_{1\leq i\leq c}\left\{\min\left\{\frac{d\left(c_{i},c_{j}\right)}{\max_{1\leq k\leq c}d\left(X_{k}\right)}\right\}\right\}$$

where c represents the number of clusters in the dataset; $d\left(c_{i},c_{j}\right)$ represents the distance between cluster $X_{i}$ and $X_{j}$ while $d\left(X_{k}\right)$ measures the distance between cluster $X_{k}$ members. The Dunn index is overly sensitive to noisy clusters [44].

**Gamma Index:** The Gamma index [45] is given as shown in Equation (29).

(29) $$G\left(C\right)=\frac{\sum_{c_{k}\in C}\sum_{x_{i},x_{j}\in c_{k}}dl\left(x_{i},x_{j}\right)}{n_{w}\left(\left(\begin{array}{c} N\\ 2 \end{array}\right)-n_{w}\right)}$$

where $dl\left(x_{i},x_{j}\right)$ represents the number of all pairs of objects in X. Gamma Index complexity is given as $O\left(\;n^{2}\;.\;d+n^{2}\;\log\;n\;\right)$. This complexity is high making it unsuitable for large datasets [4,45].

**Generalized Dunn Index (GDI):** This measures the inter-cluster and the intra-cluster distances in dataset partition [44]. The definition is given as shown in Equation (30).

(30) $$C=\frac{\min_{k\neq k^{\prime }}\delta \left(C_{k},C_{k^{\prime }\;}\right)}{\max_{k}\Delta \left(C_{k}\right)}$$

where δ and Δ are the measures of inter-cluster distance and intra-cluster distance respectively and $1\leq k\leq K\;and\;1\leq k^{\prime }\leq K$. The Generalized Dunn Index has a complexity that is the same as the Dunn index, that is, $O\left(\;n^{2}\right)\text{.}$ The quadratic complexity of the GDI makes it computationally intensive for large datasets [44,51,73].

**Ksq_DetW index:** This is also written as $K^{2}\left|W\right|$ [74]. The $K^{2}\left|W\right|$ analysis the determinant of the within cluster scatter matrix W to evaluates clusters’ compactness. The definition is given as in represented in Equation (31).

(31) $$C=K^{2}\;\det\;\left(WG\right)$$

where WG is the matrices of the individual cluster. The computational complexity for the Ksq_DetW index is $O\left(n\;.\;d^{2}+d^{3}\right)$. This is the same with the Det Ratio index. For high-dimensional data, the $d^{3}$ dominates the complexity making it computationally inefficient [27,71].

**G-plus index:** The G-plus index examines the rank-order relationship of inter- and intra-cluster distances to evaluate the quality of a clustering. It uses the concept of concordant and discordant pairs. If the intra-cluster distances of a pair of clusters are smaller than the inter-cluster distances, the pair is said to be concordant, The definition of the G-plus index [75] is given as shown in Equation (32).

(32) $$G+=\frac{2S^{-}}{N_{T}\left(N_{T}-1\right)}$$

The computational complexity is given as $O\left({n\;}^{2}.n+n^{2}\;\log\;n\right)$. The quadratic complexity due to the computation and ranking of the pairwise distances makes the G-plus index computationally intensive for large datasets.

**Log_Det_Ratio index:** Log_Det_Ratio index [76] is the Det_Ratio logarithmic version. Log_Det_Ratio index determines the quality of clusters using the log determinants of the ratio of the between-cluster scatter matrix and the within-cluster scatter matrix. It is defined in Equation (33).

(33) $$C=N\;\log\left(\frac{\det\;\left(T\right)}{\det\;\left(WG\right)}\right)$$

The computational complexity is given as $O\left(n\;.\;d^{2}+d^{3}\right)$ [4,71].

**McClain-Rao index:** McClain-Rao index [77] finds the average of the ratio of within-cluster and between-cluster distances. It has a quadratic computational complexity with respect to the number of data objects and it is given as $O\left({n\;}^{2}.d\;\right)$ The minimum value gives the best partition. The definition of the McClain-Rao index is given as presented in Equation (34).

(34) $$C=\frac{N_{B}S_{w}}{N_{w}S_{B}}$$

**Log_SS_Ratio index:** This Log_SS_Ratio index [78] measures the ratio of the traces of matrices BG and WG. It compares the within-cluster sum of square to between-cluster sum of square to evaluates how compact and well-separated the clusters are by taking the logarithm of the ratio between these two measures. It is defined in Equation (35).

(35) $$C=\log\left(\frac{BGSS}{WGSS}\right)$$

It has a computational complexity of O(n.d) [27,79].

**Negentropy Increment:** Negentropy Increment [80] measures the normality of clusters instead of the intra-cluster distances and inter-cluster distances. It evaluates the quality of clusters by calculating the negentropy (the distance of a distribution from Gaussian) of the dataset before and after clustering. It is defined as shown in Equation (36).

(36) $$NI\left(C\right)=\frac{1}{2}\sum_{c_{k}\in C}p\left(c_{k}\right)\log\;|\sum_{c_{k}}|-1/2\;\log|\sum_{X}|-\sum_{c_{k}\in C}p\left(c_{k}\right)\log p\left(c_{k}\right)$$

The computational complexity is given as O(n.d), [71,81].

**NIVA index:** The NIVA (Normalized-Intra-cluster and Variance distance) index measures the balance between average intra-cluster distance and the variation in inter-cluster distances to assess the quality of clusters. The definition of the NIVA index [82] is given in Equation (37).

(37) $$NIVA\left(C\right)=\frac{Compac\;\left(C\right)}{SepxG\left(C\right)}$$

SepxG(C) and Compac(C) represent the average separability and average compactness of the cluster C respectively. The NIVA index has a computational complexity of $O\left(n^{2}\;.d\;\right)$ [16,71].

**OS-index:** The Optimal Stability Index (OS-index) [83] evaluates clustering quality by assessing the stability of the clusters based on the compactness within clusters and the separation between clusters. It is given in Equation (38).

(38) $$OS\left(C\right)=\frac{\sum_{c_{k}\in C}\sum_{x_{i}\in c_{k}}OV\left(x_{i},c_{k}\right)}{\sum_{c_{k}\in C}10/\left|c_{k}\right|\sum \max_{x_{i}\in c_{k}}\left(0.1|c_{k}|\right)\left\{d_{e}\left(x_{i}\bar{c_{k}}\right)\right\}}$$

It has a quadratic computational complexity of $O\left(n^{2}\right)$ making it computationally expensive for large datasets [84].

**The Pakhira–Bandyopadhyay–Maulik (PBM) index:** The PBM index [17] is also called the *I* index. It finds the distance between the points and their barycentre as well as the distances between the barycentre. The acronym PBM is derived from the initials of the author's names. The index is defined as illustrated in Equations (39), (40), (41), (42).

(39) $$C={\left(\frac{1}{k}\times \frac{E_{T}}{E_{W}}\times D_{B}\right)}^{2}$$

where

(40) $$D_{B}=\max_{k<k^{\prime }}d\left(G^{\left\{k\right\}},G^{\left\{k^{\prime }\right\}}\right)$$

and

(41) $$E_{w}=\sum_{k=1}^{K}\sum_{i\in I_{k}}d\left(M_{i},G^{\left\{k\right\}}\right)$$

and

(42) $$E_{T}=\sum_{i=1}^{N}d\left(M_{i},G\right)$$

Three basic factors are considered in the PBM index: comparison between the total within-cluster dispersion and the total scatter of the dataset as a single cluster after partitioning; the maximum distance between cluster centroids and the inverse of the number of clusters. The computational complexity of PBM is given as $O\left(n\;.k.d+k^{2}\right)$.

**Point-Biserial index:** The Point-Biserial index [85] is a correlation-based clustering validity measure that finds the pairwise distance between data points within and between clusters. It has a computational complexity of $O\left(\;n^{2}\right)$ which makes it computationally inefficient for large datasets. The definition of Point-Biserial index is given as shown in Equations (43), (44).

(43) $$C=s_{n}\times r_{pb}\;\left(A,B\right)=\left(\frac{S_{W}}{N_{W}}-\frac{S_{B}}{N_{B}}\right)\frac{\sqrt{N_{W}N_{B}}}{N_{T}}$$

where:

(44) $$r_{pb}\;\left(A,B\right)=\frac{M_{A_{1}}-M_{A_{0}}}{S_{n}}\sqrt{\frac{n_{A_{0}}n_{A_{1}}}{n^{2}}}$$

$M_{A_{0}}$ represents the mean inter-cluster distance, $M_{A_{1}}$ represents the mean intra-cluster distance, the standard deviation of A is given as $s_{n}$ while $n_{A_{0}},n_{A_{1}}$ represents each group's number of elements. The distance between pairs of cluster points is represented by Set. If a pair of points are in different clusters, the value of B is 0 and if otherwise, the value is 1.

**Ratkowsky-Lance index:** The Ratkowsky-Lance index [86] is a centroid-based cluster validity index that calculates the sum-of-squares distances between data points and cluster centroids. It has an approximately O(n.k) computational complexity which makes it computationally feasible for small and medium-sized datasets. However, it becomes computationally expensive as the number of data points and number of clusters The definition for the Ratkowsky-Lance index is given in Equations (45), (46).

(45) $$C=\sqrt{\frac{\bar{R}}{K}=\frac{\bar{c}}{\sqrt{k}}}$$

where:

(46) $${\bar{c}}^{2}=\bar{R}=\frac{1}{p}\sum_{j=1}^{p}\frac{{BGSS}_{j}}{{TSS}_{j}}$$

The ${BGSS}_{j}$ represents the matrix BG diagonal term.

**Ray-Turi index:** The definition for the Ray-Turi index [87] is given in Equation (47).

(47) $$C=\frac{1}{N}\frac{WGSS}{\min_{k<k^{\prime }}\Delta_{kk^{\prime }}^{2}}$$

The numerator represents the mean squared distance of all points from the barycentre of their respective clusters while the denominator represents the clusters’ minimum squared distance from each other. It also has an approximate computational complexity of O(n.k) which scales up with larger datasets making it unsuitable for big data clustering applications.

**Root-mean-square standard deviation(RMSSTD):** The Root-mean-square standard deviation [50] measures the square root of all the attributes’ variance used in the clustering. By this, the RMSSTD measures the homogeneity of clusters in datasets. It also has a computational complexity of O(n.k). The definition is given in Equation (48).

(48) $$RMSSTD=\left[\frac{\sum_{\begin{array}{c} i=1\ldots nc\\ j=1\ldots v \end{array}}\sum_{k=1}^{n_{ij}}{\left(x_{k}-\bar{x_{k}}\right)}^{2}}{\sum_{\begin{array}{c} i=1\ldots nc\\ j=1\ldots v \end{array}}\left(n_{ij}-1\right)}\right]$$

**R-squared index(RS):** The definition of the R-squared index [88] is given in Equation (49).

(49) $$RS=\frac{{SS}_{b}}{{SS}_{t}}=\frac{{SS}_{t}-{SS}_{w}}{{SS}_{t}}$$

It measures the degree of dissimilarity between clusters by calculating the total variance across all data points and within-cluster variance which typically yields a computational complexity of O(n.k). It is computationally expensive for large datasets, especially for high-dimensional ones.

**Scatter Criteria:** The Scatter Criteria index measures the quality of a clustering solution by evaluating the dispersion of data points within a cluster and dispersion between clusters using scatter matrices. The total of the two scatter matrices captures the overall variance in the datasets. The computational complexity of the Scatter Criteria index is given as $O\left(\;n\;.\;d^{2}\right)$. It is computationally expensive for large-scale or high-dimensional datasets. The definition for Scatter Criteria [89] is given in Equation (50).

(50) $$S_{k}=\sum_{x\in C_{k}}\left(x-\mu_{k}\right){\left(x-\mu_{k}\right)}^{T}$$

**Score function:** The Score function [90] estimates cluster centroids ‘distances from the global centroids to evaluate the dispersion of clusters from each other. It also evaluates the clusters’ degree of closeness by measuring the distance between the data objects and their respective cluster centroids. It has a computational complexity of $O\left(\;n^{2}\right)$ and it typically scales quadratically with the number of data points. The definition for the score function index is given shown in Equations (51), (52), (53).

(51) $$SF\left(C\right)=1-\frac{1}{e^{e^{bdc\left(C\right)}+wcd\left(C\right)}}$$

where:

(52) $$bdc\left(C\right)=\frac{\sum_{c_{k}\in C}\left|c_{k}\right|d_{e}\left(\bar{c_{k},X}\right)}{N\times K}$$

and

(53) $$wcd\left(C\right)=\sum_{c_{k}\in C}\frac{1}{\left|c_{k}\right|}\sum_{x_{i}\in c_{k}}d_{e}\bar{x_{i},\left(c_{k}\right)}$$

**Scott-Symons index:** In Scott-Symons index [76], the weighted sum of the variance-covariance matrix's determinant for each cluster is evaluated. It also has a computational complexity of $O\left(\;n^{2}\right)$ making it computational inefficient for large-scale and high dimensional datasets. The definition is given in Equation (54).

(54) $$C=\sum_{k=1}^{K}n_{k}\;\log\det\left(\frac{{WG}^{\left\{k\right\}}}{n_{k}}\right)$$

where:

${WG}^{\left\{k\right\}}$ represents the matrices and the matrices’ determinants are positive.

**SD validity index:** The SD validity index [4] evaluates the mean of intra-cluster and inter-cluster scattering. The SD validity index is defined in Equations (55), (56), (57).

(55) $$SD\left(n_{c}\right)=a.Scat\left(n_{c}\right)+Dis\left(n_{c}\right)$$

where:

(56) $$Scat\left(n_{c}\right)=\frac{\frac{1}{n_{c}}\sum_{i=1}^{n_{c}}\left\|\sigma \left(v_{i}\right)\right\|}{\left\|\sigma \left(X\right)\right\|}$$

and

(57) $$Dis\left(n_{c}\right)=\frac{D_{\max}}{D_{\min}}\sum_{k=1}^{n_{c}}{\left(\sum_{z=1}^{n_{c}}\left\|v_{k}-v_{z}\right\|\right)}^{-1}$$

The SD validity index is a summation-type index. It combines the cluster compactness and separation measures in an additive way. The $Scat\left(n_{c}\right)$ is the mean of the normalized variances within the clusters while the $Dis\left(n_{c}\right)$ represents the total separation between the clusters. The SD index has a computational complexity of $O\left(\;{n\;.k.d+k}^{2}\;.\;d\;\right)$. It is computationally expensive for large datasets and high-dimensional data. S_Dbw [5] is a variant of the SD validity index that uses the density of objects in between two clusters replacing the total separation of the SD validity index and also removing the weighting factor a. Other variants of the SD validity index includes Vsv1, Vsv2 [[91], [92], [93]].

**Silhouette index:** The Silhouette index [94], requires that information about separation and compactness of at least two clusters must be known. In evaluating cluster validity using the Silhouette index, the index assigns the silhouette width, s(i)=(i=1,…,m) to the ith entity of a given cluster $X_{j}\left(j=1,\ldots c\right)$ . This is an estimate of the degree of probability that the ith sample belongs to the cluster $X_{j}$. The definition for the index is given in Equation (58).

(58) $$s\left(i\right)=\frac{\left(b\left(i\right)-a\left(i\right)\right)}{\mathrm{Max}\left\{a\left(i\right),b\left(i\right)\right\}}$$

where the mean distance between other entities in the cluster $X_{j}$ and the ith entity in the same cluster is represented by a(i) while the minimum mean distance between the ith and all the entities clustered in $X_{k}\left(k=1,\ldots ,c;k\neq j\right)$ is represented by b(i) The width of the silhouette is obtained using the normalized difference between an object's distance to the nearest object in another cluster in its neighbourhood and the mean distance to the other objects of the same cluster. A value of 1 is an indicator that an object is well positioned within its cluster, a value closer to 0 indicates that the object is at the borderline of two clusters, and a value closer to −1 indicates it should be assigned to the cluster in the neighbourhood. Silhouette index does not depend on the clustering algorithm that generates the data partition but only on the actual partition of the objects in its evaluation of cluster quality [94]. It is useful in improving cluster analysis results and useful in comparing clustering solutions of different clustering algorithms on the same datasets. The Silhouette's main strength is in the interpretation and validation of cluster analysis results. It is related to specific distance measures and so cannot be used for comparing with clustering results that use different distance measures. The Silhouette index has an approximate computational complexity of $O\left(\;n^{2}\right)$ which is considerably expensive for large datasets.

**Sum of squared error (SSE):** SSE is known to be among the most popular cluster validity evaluation criteria. It evaluates a given cluster's quality by considering only the clusters' cohesion. The definition of Sum of squared error is given in Equation (59).

(59) $$SSE=\sum_{k=1}^{K}\sum_{\forall x_{i}\in C_{k}}{\left\|x_{i}-\mu_{k}\right\|}^{2}$$

where the set of all entities in the cluster k while the vector means of k is given as $\mu_{k}$. The partition with the lowest SSE value is considered the best [95,96]. It has a computational complexity of O(n.k.d).

**SV-Index:** In the Symmetry-based Validity Index (SV-Index) [97], cluster separation is evaluated as a measure of distance between the nearest neighbours while cluster compactness is measured using the boundary points to the clusters’ centroids. The definition is given in Equation (60).

(60) $$SV\left(C\right)=\frac{\sum_{c_{k}\in C}\;\min_{ci\in C\backslash c_{k}}\left\{d_{e}\bar{\left(c_{k},c_{l}\right)}\right\}}{\sum_{c_{k}\in C}10/\left|c_{k}\right|\sum \max_{x_{i}\in c_{k}}\left(0.1\left|c_{k}\right|\right)\left\{d_{e}\left(x_{i}\bar{c_{k}}\right)\right\}}$$

The SV index aims at efficient validation of clusters whose sizes and densities differ widely. It is similar to Dunn's index GDI11. It measures the compactness using the mean distance of ten percent of objects that are farthest from the centroids of the cluster and measures the cluster separation using the sum of the smallest pairwise distance between the centroids of the clusters. It is usually used for identifying clusters with symmetric distribution. The SV index is adaptable for different data distributions and types because it can be computed using different distance metrics types. It has a computational complexity of $O\left(\;n^{2}\right)$.

**Sym-index:** The Symmetry index (Sym-index) [18] measures the symmetric distribution of clusters in a dataset. The Sym-index is based on the point-symmetry distance replacing the Euclidean metric synonymous with most classical cluster validity indices with point-symmetry distance in measuring objects' proximity to the cluster's centroid. It is mostly used in datasets with symmetric or ellipsoidal shape clusters. The Sym-index is given in Equation (61).

(61) $$Sym\left(C\right)=\frac{\max_{C_{k},C_{l}\in \left\{d_{e}\bar{\left(c_{k},c_{l}\right)}\right\}}}{K\sum_{c_{k\in }}\sum_{x_{i}\in c_{k}}d_{ps}^{*}\left(x_{i},c_{k}\right)}$$

The computational complexity of SV-index is given as O(n.k).

**Tau index:** This is also called the Tau coefficient. It is used in assessing the agreement or similarity between two clustering solutions. It measures the extent to which data element pairs are grouped or separated. The definition for the Tau index [92] is given in Equation (62).

(62) $$C=\frac{s^{+}-s^{-}}{\sqrt{N_{B}N_{W}\left(\frac{N_{T}\left(N_{T}-1\right)}{2}\right)}}$$

The numerator is not affected by the equality of the intra-cluster and inter-cluster distances because $s^{+}and\;s^{-}$ do not count ties. The Tau index has a quadratic computational complexity of $O\left(\;n^{2}\right)$.

**Trace_W index**: The Trace_W index gives the total dispersion of the cluster which is measured by the within-cluster sum of squares. The definition for Trace_W index [92] is given in Equation (63).

(63) C=Tr(WG)=WGSS

where WG represents the sum of all clusters while WGSS represents the within-cluster sum of squares. It is counted among the most commonly used cluster validity indices in clustering applications [92]. It performs well mostly when all the clusters have the same dispersion but performs poorly when clusters are hyper-spherical with different sizes. This is because the size of clusters is measured based on the number of objects it contains and not on the volume of space it occupies. It has an overall computational complexity of O(n.k.d). The computational intensity increases linearly as the number of data points and number of clusters increases as well as the number of dimensions.

**Trace_WiB index:** This is also called Hotelling's Trace Criterion. It measures the quality of the clustering solution based on the within-cluster matrix which it seeks to minimize while maximizing the between-cluster distance. The definition for Trace_WiB index [98] is given in Equation (64).

(64) $$C=Tr\left(WG^{-1}.BG\right)$$

The computational complexity of the Trace_WiB index is given as O(n.k.d).

**Wemmert-Gancarski index**: The Wemmert-Gancarski index evaluates the weighted average of all clusters’ quantities $\left(J_{k}\right)$ . The definition of the Wemmert-Gancarski index is given in Equations (65), (66), (67).

(65) $$C-\frac{1}{N}\sum_{k=1}^{K}\max\left\{0,n_{k}-\sum_{i\in I_{k}}R\left(M_{i}\right)\right\}$$

where M is an element in the cluster $C_{k}$,

(66) $$J_{k}=\max\left\{0,n_{k}-\sum_{i\in I_{k}}R\left(M_{i}\right)\right\}$$

and

(67) $$R\left(M\right)=\frac{\left\|M-G^{\left\{k\right\}}\right\|}{\min_{k^{\prime }\neq k}\left\|M-G^{\left\{k^{\prime }\right\}}\right\|}$$

The Wemmert-Gancarski index measures the number of objects closer to the centroid of their cluster than to other clusters' centroids [26]. proposed a variant to the Wemmert-Gancarski index using the idea of the Silhouette index defining each object's cluster membership score based on a comparison of each object's distance from its cluster's centroid and its minimum distance from the centroid of other clusters. The Wemmert-Gancarski index has a computational complexity of O(n.k).

**Xie-Beni index:** The Xie-Beni index [99] finds the ratio of the mean quadratic error and the minimum of the squared distances between the points in the cluster. The definition is given in Equations (68), (69).

(68) $$C=\frac{1}{N}\;\frac{WGSS}{\min_{k<k^{\prime }}\delta_{1}{\left(C_{k},C_{k^{\prime }}\right)}^{2}}$$

where:

(69) $$\delta_{1}{\left(C_{k},C_{k^{\prime }}\right)}^{2}=\min_{\begin{array}{c} i=I_{k}\\ j=I_{k^{\prime }} \end{array}}d\left(M_{i},M_{j}\right)$$

In the Xie-Beni index, the cluster cohesion is measured using the global mean squared distance of objects from the centroid of their cluster while the inter-cluster separation is measured using the minimum squared distance between pairs of clusters [26]. Xie-Beni index is reported as demonstrating a monotonic decreasing tendency as the cluster number gets larger and near the number of objects. Variants of Xie-Beni include Ray-Turi, Kw index, Tang index, and XB2. The XB2 variant uses the maximum cluster variance in place of the global mean of cluster compactness to avoid the general tendency common with averaging which tends to hide the unnecessary merging of clusters effect. It has a computational complexity of O(n.k).

Algorithm listing 1 presents a high-level pseudo-code of the algorithm for clustering validity indices as would be incorporated into any metaheuristic methods with or without any further modification.

**Table:** 

**Algorithm 1:** Pseudocode for Generic Cluster Validity Indices	
**Input:** **Output:** **1.** **2:** **3:** **4:** **5:** **6:** **7:** **8:** **9:** **10:** **11:** **12:** **13:** **14:** **15** **16**: **17:** **18:** **19:** **20:** **21:**	Array {$x_{1},x_{2},x_{3},.\;x_{n}$}//Dataset to be clusters k//Number of required clusters $CC=\left(\;{cc}_{1},{cc}_{2},{cc}_{3},\ldots {cc}_{k}\;\}\right)$//Cluster centroids Cluster Validity Index Value //Initialize Parameters $X=\left(\;x_{1},x_{2},x_{3},\ldots x_{n}\}\right)$ $CC=\left(\;{cc}_{1},{cc}_{2},{cc}_{3},\ldots {cc}_{k}\;\}\right)$ MinInterClust = minItc MaxInterClust = MaxItc //Compute Intra-cluster distance for i=1tok do Compute intra-cluster distance Update intra-cluster distance end i //Compute Inter-cluster distance for i = 1 to k for j = i+1 to k Compute inter-cluster distance Update inter-cluster distance end j end i //Compute Cluster Validity Index Value Use the CVI function to compute the cluster validity index value Output Cluster Validity Index Value End

## 5 Review findings

In this section, a review of the selected articles with reference to the cluster validity index used as a fitness function in automatic clustering is presented with an emphasis on the performances of the CVIs. In Ref. [124], an automatic metaheuristic-based clustering algorithm using Particle swarm optimization is reported. The authors used Dunn's, Turi and the S_Dbw validity indices. Their report shows that the Turi validity index performed better than the other two validity indices. The authors in Ref. [125] used the Sum of Square Error, Variance Ratio Criterion, and Davies Bouldin index in evaluating their automatic clustering algorithm based on the combinatorial Particle swarm optimization metaheuristic algorithm.

In the automatic clustering algorithm reported by Ref. [126], the Calinski-Harabasz index and Rand Index were used as the cluster validity index. The Turi index was employed by Ref. [127]in the improved Particle swarm optimization automatic clustering algorithm. They observed that obtaining a better Turi index value does not ensure higher accuracy. Their suggestion is to use another validity index if the main concern is accuracy. Moreover, they also observed that the similarity measurement significantly influenced the results obtained.

In [128], variance [129] and connectivity [130] were used in their multi-objective immunized PSO automatic clustering algorithm. A kernel-induced similarity measure was adopted in the CS measure by Ref. [131] for automatic clustering based on the Multi-Elitist Particle swarm optimization algorithm instead of the usual sum-of-squares distance as a kernelized distance metric in the CS cluster validity index. The CS measure was noted as more efficient in handling clusters of different sizes and/or densities compared with other popular validity indices due to high computational loads as the number of clusters and datasets increases.

In the automatic clustering using Multi-objective Particle Swarm and Simulated Annealing algorithms reported in Ref. [132], three cluster validity indices were used: the DB index, Sym-index, and Conn-index using the Euclidean distance for cluster connectedness, symmetry for total cluster symmetry and cluster connectedness in each of the CVIs respectively. The adoption of the three CVIs in the multi-objective function helped in the detection of clusters in datasets with various shapes as well as overlapping and non-convex datasets. In the work of [37], two CVIs were used, the DB index and the CS measure. They observed that the two CVIs were not efficient with datasets that had overlapping clusters.

In [133], the *I* cluster validity index was used in their differential evolution automatic clustering algorithm using the cluster number oscillation method. In the Differential Evolution Fuzzy clustering for Automatic cluster evolution proposed by Ref. [134], the Xie-Beni index was used as the CVIs for the automatic cluster evolution algorithm. In Ref. [135], the *I* index was added to the Xie-Beni index for the cluster validity evaluation of their modified Differential Evolution-based automatic clustering algorithm. In Ref. [136], the Xie-Beni index and Silhouette index were used in the multi-objective Differential Evolution automatic clustering algorithms. The Xie-Beni index was also adopted by Ref. [137] in their automatic clustering using the synergy of GA and multi-objective DE.

The authors [138] opined that the effectiveness of automatic fuzzy clustering methods is dependent on the selection of the validity indexes. Moreover, using a single-objective function may not yield satisfactory results in real-world applications like remote sensing images due to the complexity involved in such applications. They used the Xie-Beni index and $J_{m}$ in their proposed adaptive multi-objective DE for automatic clustering in remote sensing imagery. According to them, optimizing several validity measures simultaneously is necessary to adequately cluster datasets with varying characteristics.

In automatic clustering using genetic algorithms, Liu, Wu, and Shen [36] employed the DB index to evaluate the automatic clustering result. Their observation was that it is difficult to use one CVI to deal with different datasets. They proposed to use another validity index such as the PBM index for their future research. In Ref. [139], the authors adopted the CH index in their two-stage genetic algorithm for automatic clustering. Their report further noted the challenges in some of the existing cluster validity – the computational heaviness and difficulty with noisy data observed with the Dunn index [90] but only useful for identifying clean clusters in datasets whose sizes are not more than hundreds of points; the DB index's inability to accommodate datasets with overlapping clusters though it gives good results in datasets with distinct clusters; inapplicability of Silhouette index to handle datasets with sub-cluster because it is only able to identify the first choice and the PBM index's dependency on user-specified parameters.

The Xie-Beni index, Sum of Square Error, and COSEC fitness function were used by Ref. [140] in their hybrid clustering technique based on genetic algorithms with K-means. The authors in Ref. [141] used the VI index on account of its satisfactory performance as reported in Refs. [142,143]. In Ref. [144], the CS_kernel_ measure was used in evaluating the performance of their proposed automatic clustering algorithm. A kernel function replaced the conventional Euclidean distance for efficiently handling datasets with different scales and densities. The use of the kernel function is good with complicated and linearly inseparable datasets. The authors [145] adopted the VI value as the cluster validity index for their automatic clustering algorithm based on artificial bee colony for customer segmentation.

The PBM index was used by Ref. [146] for the fitness function for their proposed dynamic parameter harmony search optimization algorithm (AC-DPHS)automatic clustering algorithm. PBM was compared with the DB index and XB index and reported to exhibit better performance in terms of the optimal number of clusters and the lower computational time. However, the effect of the clustering quality for higher dimensional datasets is not too obvious and a suggestion for better clustering validity indexes was suggested for higher dimensional datasets.

### 5.1 Analysis of the CVIs usage in automatic clustering algorithms

**Fig. 2:**
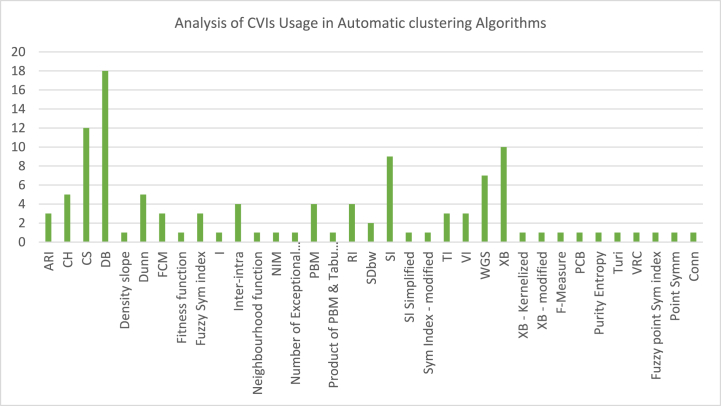
Analysis of reviewed literature for cluster validation.

The analysis of the reviewed articles regarding the indices used for cluster validation is presented in Fig. 2. The highest number of articles used the Davies-Boulding index for cluster validation followed by CS, SI, and Xie-Beni indices respectively. The strengths and weaknesses of the CVIs have been summarized in Table 3 as obtained from the reviewed articles.

### 5.2 Factors affecting the performances of cluster validity indices

Cluster validity indices are measured based on the relationship between cluster characteristics such as cluster cohesion, cluster separation, cluster symmetry, and connectedness [1]. These basic cluster characteristics are determined using some proximity metrics such as the Euclidean distance, the Cosine distance, the maximum edge distance, etc. The proximity measure adopted in any cluster validity index determines the shape of the clusters that can be identified. For instance, the use of Euclidean distance identifies spherically shaped clusters while the maximum edge distance is good at discovering irregular-shaped clusters. The Cosine distance is employed mostly when priority is given to discovering the orientation between patterns rather than their magnitude.

In determining the closeness or similarity of objects in a dataset, the distance measure used has a considerable effect on how the data objects are clustered [100]. Cluster validity indices that use traditional variability criteria(variance, separation, density, and continuity) for cluster validation are not efficient when handling arbitrarily shaped clusters [4]. Validity indices that do not use average values in their evaluation metric perform better in validating clusters of different densities and sizes. According to Ref. [31], the standard measure of interest, that is, distance, is the least reliable measure for cluster validation for clusters of volumetric cloud forms. In cluster validity indices that use Euclidean distance, scaling of various dimensions also affects the clustering patterns.

Moreover, from the study conducted by Ref. [147], the cluster validity indices' reliability/performance varies to the clustering method, the data structure as well as the clustering objective. According to Ref. [5], cluster overlap, experimental factors, and the presence of noise have an impact on cluster validation indices. According to Ref. [5], most CVIs demonstrate better results with fewer clusters. Jiang et al. reported that distance among features becomes meaningless in high dimensional datasets more so in data gene expression where the overall shape of the gene expression patterns is more important [148]. Hence the Pearson's correlation coefficient is used in measuring the similarities in the shapes of gene expression patterns.

It is generally concluded that no cluster validity index provides consistent results for different clustering algorithms thus emphasizing the fact that none perform better than others. It is recommended that many validation indexes should be employed to determine the best-performing one for various datasets.

### 5.3 Application areas common cluster validity indices

#### 5.3.1 Web Usage

Web intelligence is the general term for describing the research and application of information technology and machine learning focussing on the Web platform. Web intelligence applications include Web document clustering, classification of online text, Web usage profiling, e-commerce web recommender, and other tasks involving knowledge discovery [149]. Web Usage data are often unstructured and characterised by complex attributes. They are usually generated from Web activities dynamically and asynchronously. Clustering plays an important role in mining and extracting knowledge from Web intelligence data and related applications.

Clustering web documents organizes knowledge, enhances search engine results, and enhances web crawling [150]. Metaheuristic optimization approaches have been applied in web document clustering due to the high dimensionality and orthogonality characteristics of web documents. The authors [150] suggest Entropy-based measures and cluster cohesiveness measures as fitness functions for web document clustering. In text classification, unstructured sets of documents are partitioned into their respective category based on their content [151]. Due to the exponential increase in the growth of information over the internet, there is a need for automatic classification of text documents. The area of application of text classification includes topic tracking, spam filtering, sentiment analysis, web page classification, and email routing.

The authors [149] investigated and reported the efficacy of the application of nature-inspired optimization algorithms such as the Fireflies, Cuckoos, Wolves, and Bats for Web Intelligence data clustering. The performance of the clustering algorithms was measured using the inter-cluster distance and intra-cluster distance. A Fuzzy-based Recommender System for Web Users was proposed by Ref. [152] which uses an algorithm to provide acceptable data clusters without prior knowledge of the initial clusters. Similarity and distance measures were used in calculating the match score for the recommender system.

#### 5.3.2 Speech processing

Speech provides a natural way of communication among humans. The study and processing methods of speech signals are referred to as speech processing. It includes speech-coding algorithms, speech recognition, speech synthesis, and other aspects of speech processing. The speech-coding algorithms provide effective and efficient voice communication and storage. The ability of computers to understand human language and follow human voice commands is made possible through speech recognition. The synthesis creates a platform for interactive systems that correspond to humans with natural voices [153].

According to Ref. [154], one of the most successful yet fundamental techniques in speech recognition, speech coding, image coding speaker recognition, and speech synthesis is Vector Quantization. The techniques of Vector Quantization are regarded as data clustering methods [155]. It involves compressing voice data for transmission or storage while retaining the data fidelity. A set of k-dimensional data vectors is encoded by the VQ encoder with a much smaller subset called a codebook.

The authors [155] used the Linde-Buzo-Gray (LBG) algorithm to automatically generate initial centroids using a splitting procedure. The LBG algorithm is a local optimization procedure and uses various approaches for its optimization task. The author used the directed search binary-splitting approach for the vector quantization. In Ref. [156], automatic clustering was applied to find an appropriate number of clusters in the application of the clustering method for capturing phonetic classification to establish the reliability of automatic clustering in phonetic classification. The Davies Boulding and *I* validity indices were employed to validate the quality of the generated clusters [157]. proposed a new spectral clustering algorithm that is based on minimizing a cost function built on measures of error between a solution of the spectral relaxation of a minimum cut problem and a given partition. This spectral clustering was used as a learning algorithm in speech separation problems.

In [158], a method for automatic clustering of similar units for unit selection in speech synthesis is presented. The distance between two units is measured using acoustic measure which gives the mean weighted distance between units with the shorter unit linear interpolated to the longer unit. The problem of automatic classification of speech data was addressed by authors in Ref. [159] without clearly defining the categories to characterize different speaking styles. They proposed an x-means clustering that clusters the data based on a pre-defined distance measurement that is formulated using a human perception-based weighted distance.

#### 5.3.3 Onset and progression of disease in medical science

The health sector is regarded as one of the primary sectors that has a general impact on the members of the public. Therefore, the improvement of the healthcare sector alongside contemporary society's development is very important. Diseases pose a serious threat to public health across the globe. Analysis of healthcare data has assisted patients, health officials, and healthcare communities in the early detection of many diseases [160]. Access to complete medical data obtained from patterns extracted from healthcare data has assisted in improved medical diagnosis and treatment. A huge number of medical images are generated daily. Analysis of these medical images using image segmentation to identify regions of interest has assisted in extracting important features that aid in the diagnosis of diseases. Clustering has been used as an important tool in addressing the challenge of analysing big image data.

In the work of [161], the desired cluster numbers were identified using learning vector quantization in their proposed automated system for retinal image analysis for the diagnosis of eye diseases. The authors [162] adopted an automatic clustering method for COVID-19 CT image segmentation to assist in diagnosing the disease. They used the generalized extreme value (GEV) to improve the density peak clustering (DPC) in finding the optimal number of clustering centres in their proposed model. The structural similarity index, peak signal-to-noise ratio, and entropy were used to measure the performance of the proposed algorithm.

In [163], the Mean Shift clustering method was used to automatically identify a cluster using kernel density estimation of a predetermined feature space for functional Magnetic Resonance Imaging (fMRI) used in the identification of activations regions in the brain. The authors [164] solved the problem of intensity inhomogeneity and the associated challenges of initialization and configuration of controlling parameters in medical image segmentation. They proposed a method that integrates a variation of fuzzy clustering with a local region-based level set method to automatically determine the region of interest in the image segmentation. The fuzzy local similarity measure was applied to ensure robustness against noise and for image detail preservation.

In [165], a semi-supervised clustering technique based on multi-objective optimization based on simulated annealing was proposed and applied for the automatic segmentation of MR brain images. Three cluster validity indices (Sym-index, I-index, and Minkowski index) were used as the objective functions for the system. The Sym-index used the symmetry distance metric while the I-index used the Euclidean distance metric. A hybrid automatic clustering algorithm proposed by Ref. [166] was used in the cluster analysis of prostate cancer data. Their proposed automatic clustering algorithm combined automatic kernel clustering with bee colony optimization, and it used the ${CS}_{kernel}$ index as the objective function in optimization with the aim of efficient handling of datasets with different scales and densities.

Automatic clustering has been used in deciphering hidden patterns in gene expression data. In the review work of [167] regarding the application of clustering algorithms to gene expression data, several automatic clustering algorithms were reported. In Ref. [39], a multi-objective clustering technique was proposed which automatically partitioned gene expression data into an appropriate cluster number. Three objective functions were used simultaneously for the detection of appropriate cluster numbers and optimum clustering of the gene expression data. Other automatic clustering algorithms used for gene expression data include [39,130].

The authors [168] propose an automatic clustering algorithm for medical big data clustering based on a modified Immune Evolutionary Algorithm. The objective function f based on the FCM objective function J was adopted for the optimization process.

#### 5.3.4 Image processing and image segmentation

Image processing involves the application of an extensive range of possible computational operations to an image for knowledge discovery. Image segmentation is an aspect of image processing that involves exhaustive homogeneous partitioning of an image based on some image property. Automatic clustering methods have been applied in solving problems relating to image processing and segmentation. Articles reporting applications of automatic clustering methods for image processing and segmentation include [38,162,[169], [170], [171], [172], [173], [174]].

#### 5.3.5 Retrieval of information

Information Retrieval (IR) focuses on discovering effective computational approaches for automating document storage and retrieval [175]. It involves the process of digging out queries for multimedia information, images, or specific text from web content. The information retrieval techniques find applications in a wide range of fields such as research publications, e-commerce, academics, clinical decision support, etc [176]. The adoption of massive online digital content in this era of digitization has made information retrieval cumbersome and more complex. Evolutionary-based approaches and swarm intelligence approaches transform IR problems into optimization problems using the collection of documents as a space of solutions [177].

The authors [176] proposed a swarm-optimized cluster-based framework of information retrieval using the K-Flock clustering algorithm. To evaluate the performance of the clusters, the modified silhouette coefficient [178] index measure was adopted [179]. Augmented user's original query for information retrieval through a query expansion process based on a Fire-fly algorithm-based approach. The Firefly algorithm was used to find the best-expanded query among a set of expanded query candidates for effective query expansion retrieval while maintaining low computational complexity. The inverted indexes of the terms in the expanded query were used to compute the scores for each document with the best score considered as the fitness value for the expanded query. Other works reported by these authors on automatic clustering for DIR problems can be found in Refs. [[180], [181], [182]].

In [183], automatic query expansion using cuckoo search and accelerated particle swarm optimization techniques for IR problems was proposed. The authors used the same fitness function as the one used by Ref. [179]. Other work relating to automatic query expansion is reported in Refs. [184,185]. The authors [186] proposed the use of Cellular Automata to improve the quality of clustering for information retrieval. In Ref. [187] a relevance and interface-driven clustering for visual information retrieval is proposed. Their proposed cluster algorithm automatically generates highly relevant clusters while optimizing for interface-driven desiderata for spatial, temporal, and keyword coherence and excluding the need for specification of complex distance metrics. For Automatic clustering-based IR [188], reported that the Cosine similarity measure is particularly good for text documents as a distance measure in cluster validity indexes.

The authors [189] implemented a modified firefly algorithm adapted to Intelligent Ontology and Latent Dirichlet Allocation Information Retrieval model for the enhancement of query searching time information retrieval systems. The cluster validity is based on the Semantic relevancy which is determined using the document topical strength measure. Other research reports on automatic clustering and Information retrieval include [190].

#### 5.3.6 Automotive and aviation systems

Trajectory clustering in aviation is a technique that identifies prevailing aircraft patterns. In trajectory clustering, similar trajectories or trajectory segments are identified and classified into clusters that have the potential to reveal the movements and behaviours of the corresponding objects or nodes [191]. Improving efficiency in aviation systems requires that the actual flight trajectory of aircraft is close to their ideal profile. The authors [192] proposed Trajectory clustering that uses both temporal and spatial features in approach trajectory and aircraft descent optimization based on a multi-objective perspective to minimize aircraft emission, fuel consumption, and the impact of noise.

Automatic clustering has been applied in Automatic Identification System trajectory clustering for maritime safety. It provides a theoretical basis for route planning design and management. It also strengthens the monitoring of ships dynamically and improves maritime supervision efficiently. Authors [193] proposed an automatic multi-step trajectory clustering method for robust shipboard Automatic Identification System trajectory clustering. It was used to find the customary vessel routes and detect abnormal trajectories. The authors [194] proposed a solution for anomaly detection for components of different products in the automotive industry using an automatic clustering algorithm. Six different cluster validity indexes including the Silhouette index, CH index, WB index, Sum of Square Within Clusters (SSW), Sum of Square Errors (SSE), and Sum of Square Between Clusters (SSB) were used for cluster validation.

In the work of [195], a cluster-based adaptive network fuzzy inference system tuned by Particle Swarm Optimization for the forecasting of annual automotive sales was developed. The authors in Ref. [196] Proposed an auto-tuning controller using multi-layer Particle Swarm Optimization with K-means clustering and adaptive learning strategy for Permanent magnet synchronous motor drives was proposed The proposed system uses the Square Error criterion as its fitness function. The authors [197] proposed an automotive product analysis based on automatic MP-DP-Kmeans clustering using MP similarity in place of the Euclidean distance to analyse and make the horizontal comparison of competitor products in automotive product development.

#### 5.3.7 Bioinformatics

Bioinformatics is an interdisciplinary field that mainly involves genetics and molecular biology, statistics, computer science, and mathematics. It has to do with addressing data-intensive large-scale biological problems from a computational point of view. Application of automatic clustering in Bioinformatics can either be in the form of analysing gene expression data which were generated from DNA microarray technologies or by direct clustering process on protein sequences or linear deoxyribonucleic acid (DNA) data [34,198]. Clustering of gene expression data helps in identifying patterns within datasets that relate to this domain and provides insights on natural structures inherent in biological data, understanding of gene functions, subtypes of cells, cellular processes, and gene regulations [199].

The authors in Refs. [200,201] proposed an automatic multiple kernel density clustering algorithm for handling high-dimension bioinformatic data as well as incomplete datasets in bioinformatics respectively [202]. proposed an automatic clustering algorithm for grouping brain tumour gene expression datasets based on Cuckoo search clustering and levy flight cuckoo search [203]. Hybridized Genetic Algorithm with Cuckoo search algorithm for automatic clustering of Breast Cancer dataset. The Silhouette coefficient index was utilized as an objective function for the clustering algorithm.

In [204], microarray gene expression data clustering based on a two-stage meta-heuristic algorithm that uses the concept of alpha-planes in general type-2 fuzzy sets was considered. The alpha-plane for general type-2 fuzzy c-means was used as the objective function for the clustering process. The automatic metaheuristic-based clustering was based on a Simulated Annealing optimization algorithm. Authors [205]introduced a soft computing metaheuristic framework for the automatic clustering of DNA sequences with intelligent techniques based on the Bat algorithm hybridized with the Genetic algorithm. They adopted the pulse-coupled neural network for calculating the DNA sequence similarity or dissimilarity. Their algorithm was used for clustering the expanded human oral microbiome database.

A hybrid gene selection algorithm for cancer classification was proposed by Ref. [206] based on the Bat algorithm. A minimum redundancy maximum relevancy filtering method with a Bat algorithm wrapper method was used for gene selection in the microarray dataset. An article on the soft computing methods that have been used in Bioinformatics was published by Ref. [207] stating clustering as one of the soft computing methods. He summarized some applications of sequence alignment and the soft computing methods indicating metaheuristic and swarm intelligence algorithms as the most used soft computing algorithms for sequence alignment. There is also a literature survey on population-based metaheuristic algorithms used for Gene clustering by Ref. [208] with emphasis on the application of Genetic Algorithm and Particle Swarm Optimization algorithm, their variants and hybridization.

## 6 Experimental study

This section presents the report on the experimental study carried out using eight cluster validity indices on the SOSKmeans clustering algorithm [209]. The SOSKmeans clustering algorithm is a hybrid algorithm that combines a symbiotic organism search metaheuristic algorithm with the classical K-means algorithm. It harnessed the benefits of the two algorithms for handling automatic clustering problems. The parameter setting of the algorithm is summarized in Table 4. The algorithm was executed for 200 iterations over 20 replications for each cluster validity index. The algorithm was executed using MATLAB 2018 on an Intel Dual Corei7-7600U CPU with 2.80 GHz and 15.8 GB RAM. The performance of each of the CVI was evaluated using the average best fitness value obtained for each dataset and the average computational time for convergence.

**Table 4:** SOSK-means algorithm parameter setting.

Parameter	Description	Value
Max-It	Number of iterations	200
NP	No of population	20

### 6.1 Datasets

Twelve datasets consisting of synthetic and real-life datasets with different characteristics were considered in this study. The summary of the datasets is presented in Table 5. Breast, Glass, Iris, Thyroid, Wine, and Yeast are real-life datasets that are taken to represent different domains in Engineering and Science. The remaining datasets are synthetic representing non-linearly separable datasets with arbitrary shape clusters. The Jain dataset represents complex shapes with overlapping characteristics. The compound and flame datasets are a representation of non-linearly separable clusters with different shapes and densities. Path-based, Spiral, and Two-moons datasets are a representation of arbitrarily shaped clusters with intertwined clusters with Path-based exhibiting more complex paths than the other two. The datasets are commonly used in literature for evaluating the performance of clustering algorithms on non-linearly separable data. The clustering illustration for the cluster structure of each of the datasets can be found in Ref. [209].

**Table 5:** Characteristics of the datasets.

Datasets	Dataset Types	Number of Objects	Dataset Features	Number of Clusters	References
Breast	UCI	699	9	2	[210,211]
Glass	UCI	214	9	7	[210,211]
Iris	UCI	150	4	3	[210,211]
Thyroid	UCI	215	5	2	[210,211]
Wine	UCI	178	13	3	[210,211]
Yeast	UCI	1484	8	10	[210,211]
Compound	Shape	399	2	6	[210,212]
Flame	Shape	240	2	2	[210,213]
Jain	Shape	373	2	2	[210,214]
Path-based	Shape	300	2	3	[210,215]
Spiral	Shape	312	2	2	[210,215]
Two-moons	Shape	10,000	2	2	

### 6.2 Evaluated CVIs

Eight different internal cluster validity indices were considered in this study. The CVIs include the General Dunn Index, PBM Index, CH Index, SI Index, DB Index, CS Index, Xie-Beni Index, and the Dunn-Symmetric Index. Details of these CVIs have been presented in section 4.2. The CVIs were used as an internal validity index in the metaheuristic-based automatic clustering algorithm -SOSKmeans algorithm [209] for this study. For each of the CVIs, the algorithm was executed using twenty independent runs of 200 iterations on each dataset and the result of their performances is presented in Table 6. The computation time of the various CVIs for each dataset is presented in Table 7.

**Table 6:** CVIs Average Clustering Performance on each dataset.

	Average Clustering Performance							
Dataset	GDI	S_Dbw	XB	CH	Dunn-Sym	SI	DB	CS
Breast	0.1281	0.045155	0.15874	12.9768	**0.011814**	0.700576	1.2416	1.1019
Compound	0.022873	0.009323	0.093588	22.1904	**0.002889**	0.59727	0.519386	0.77324
Flame	0.020888	0.020641	0.14963	17.3804	**0.01999**	0.318642	1.173924	1.55968
Glass	0.071372	0.001358	0.070395	4.52524	**0.004836**	0.102634	0.82578	0.02
Iris	0.027458	4.11E-05	0.128888	3.042554	**2.59E-08**	85352.2	0.84139	1.13144
Jain	0.010184	0.006578	0.12338	29.1736	**0.001478**	0.607982	0.6532	1.02611
Path-based	0.77061	0.0096	0.14029	27.5826	**0.000734**	0.684518	0.784962	0.968948
Spiral	0.017882	0.011035	0.196416	25.3902	**0.000912**	0.689684	0.80329	1.17602
Thyroid	0.030312	2.78E-08	0.054334	1.102296	**6.41E-09**	114850	0.63196	1.57881
Two-moons	0.00483	0.025071	0.111604	142.5633	**0.000878**	0.202264	0.73901	0.922632
Wine	0.222994	0.011253	0.404006	0.35299	**7.38E-06**	1197.974	1.0061	1.40034
Yeast	0.10069	0.00038	0.108704	3.39498	0.043199	**0.018009**	0.762546	0
Average	0.119016	0.011703	0.144998	24.13962	**0.007228**	16783.67	0.831929	0.971593

**Table 7:** CVIs Average Computation Time expended on each dataset.

	Average Computational Time							
Dataset	GDI	S_Dbw	XB	CH	Dunn-Sym	SI	DB	CS
Breast	6845.7	11981.4	5348.62	1935.74	14330.4	2306.78	**960.21**	13514
Compound	6115.78	5572	5458.94	1203.78	10569.4	1216.45	**881.4**	3938.8
Flame	5694.1	5675.28	4734.42	2168.2	9245.78	1175.54	**1092.9**	2451.6
Glass	4807.8	2843.78	2049.84	1202.45	4548.68	1357.866	**649.57**	2007.5
Iris	5610.06	3042	2485	1430.836	5760.98	887.272	**604.2**	1450.2
Jain	6951.2	5811.54	5338.26	1237.858	6284.62	1219.56	**1026.4**	3473.9
Path-based	1413.44	4300.84	5128.22	1413.44	10018.72	**1390.88**	1396.7	2492.4
Spiral	6587.48	5770.68	4004.32	2485.84	9916.82	1664.634	**1208.7**	2849.3
Thyroid	3692.74	1999.32	1320.84	1320.026	3337.66	1159.196	**730.35**	2282.2
Two-moons	7076	19002.6	9970.48	4134.7	29142.66667	**1236.64**	2824.1	13712
Wine	6066.26	4546.3	2625.5	1896.94	6564.62	2064.188	**654.03**	1863.2
Yeast	10953	16129.8	11775.78	7396.12	26362.66667	4775.05	**1295.6**	46894
Average	5984.46	7223	5020.02	2318.83	11340.25	1704.51	**1110.35**	8077.43

It is important to note that while internal cluster validity indices address the challenge of determining the validity of the number of clusters, metrics such as compactness and separation, associated with these indices, are employed to evaluate the quality of the clustering task. More so, cluster quality can be assessed by examining the stability of the clustering algorithm under variations in data or algorithm parameters. These steps form the basis of the experimental approach described in this study.

### 6.3 Experimental results

From Tables 6 and it can be observed that the Dunn-Sym index demonstrated superior performance in ten of the twelve datasets compared with the other CVIs followed by S_Dbw with better performance in the remaining two. The GDI followed the Dunn-Sym and S_Dbw considering its average performance compared with the rest of the CVIs. Though the Dunn-Symm demonstrated superior performance, it recorded a greater computation time compared with the other CVIs. From the experimental results, it is obvious that GDI, S_Dbw, XBI, and SI performed better on these datasets compared with the traditional DBI and CSI. The performance of the CH index could not be compared with others using the fitness value because it is a maximization technique that produces higher values.

From Table 7 showing the average computation time of the CVIs, it can be observed that the average computational time for the clustering process is lower for the traditional DBI, CS, and CH compared with the better-performing CVIs. The performance of each CVI for each of the datasets is shown in Fig. 3, Fig. 4, Fig. 5, Fig. 6, Fig. 7, Fig. 8, Fig. 9, Fig. 10, Fig. 11, Fig. 12, Fig. 13, Fig. 14 while the performance of each CVI on all the datasets is illustrated in Fig. 15, Fig. 16, Fig. 17, Fig. 18, Fig. 19, Fig. 20, Fig. 21, Fig. 22.

**Fig. 3:**
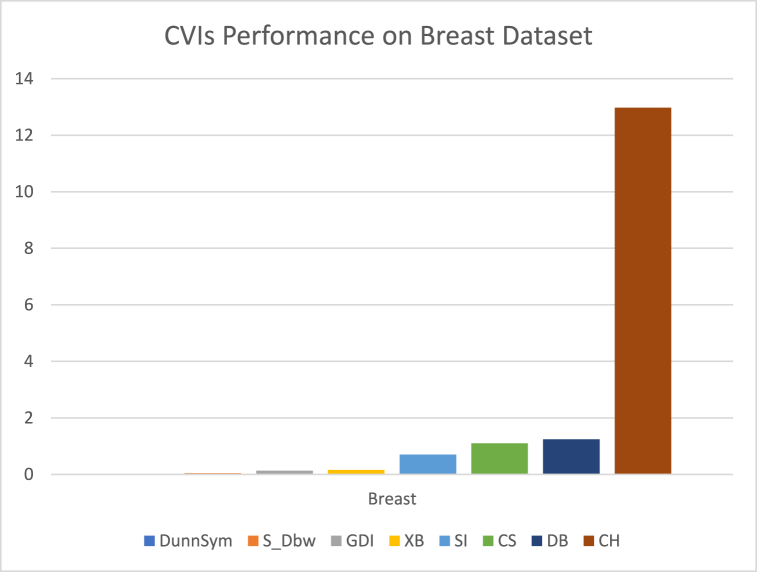
CVIs performance on Breast Dataset.

**Fig. 4:**
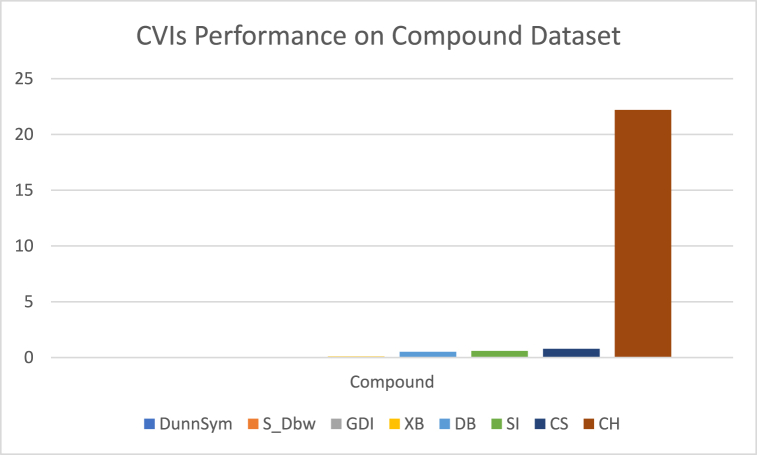
CVIs performance on compound Dataset.

**Fig. 5:**
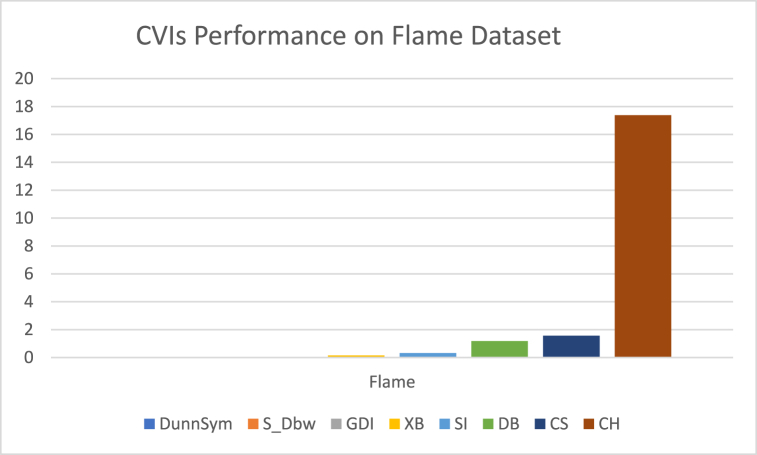
CVIs performance on flame Dataset.

**Fig. 6:**
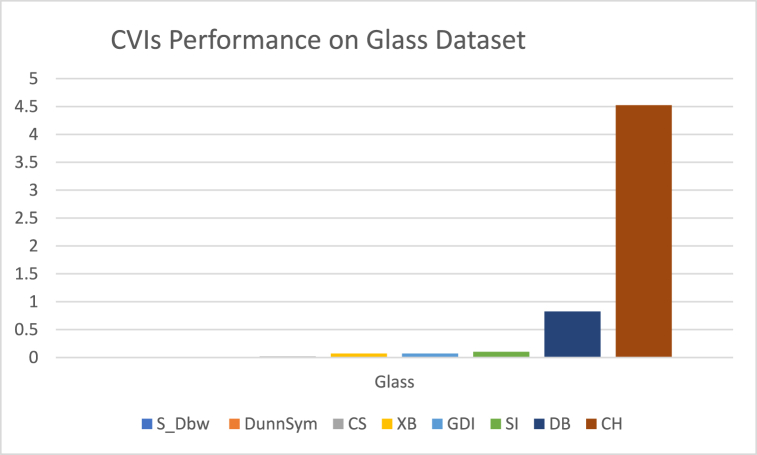
CVIs performance on Glass Dataset.

**Fig. 7:**
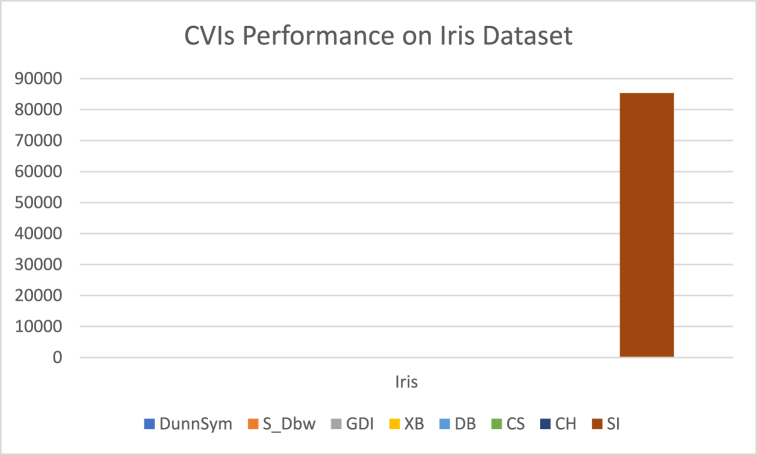
CVIs performance on Iris Dataset.

**Fig. 8:**
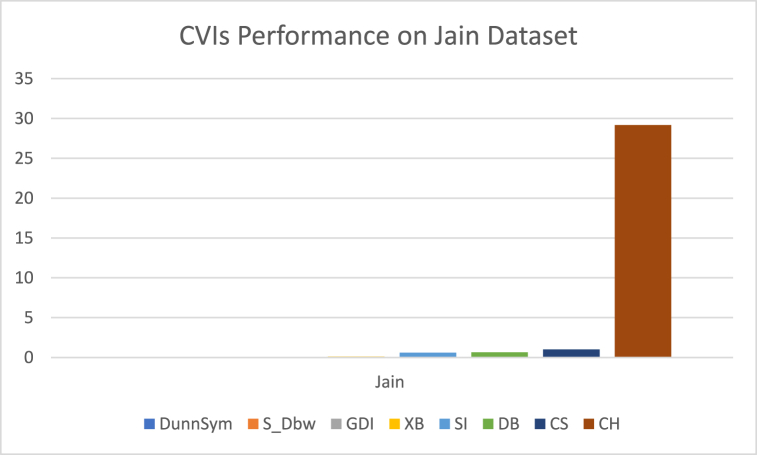
CVIs performance on Jain Dataset.

**Fig. 9:**
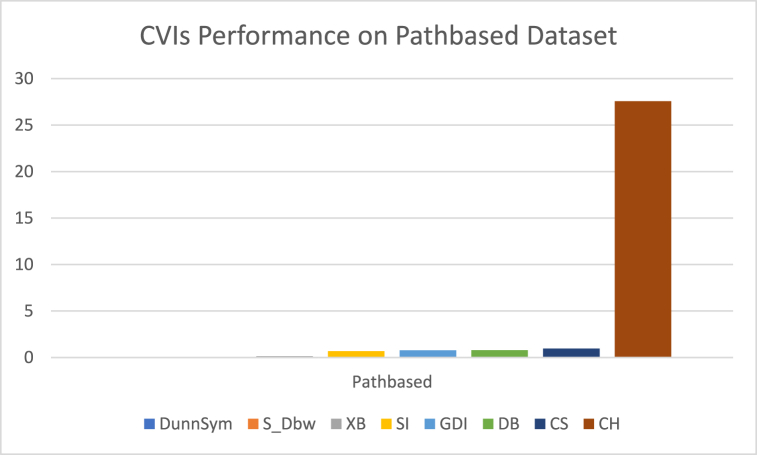
CVIs performance on path-based Dataset.

**Fig. 10:**
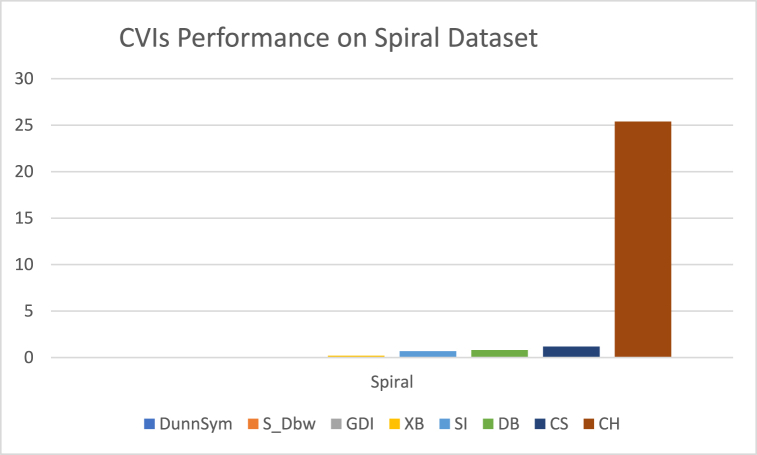
CVIs performance on Spiral Dataset.

**Fig. 11:**
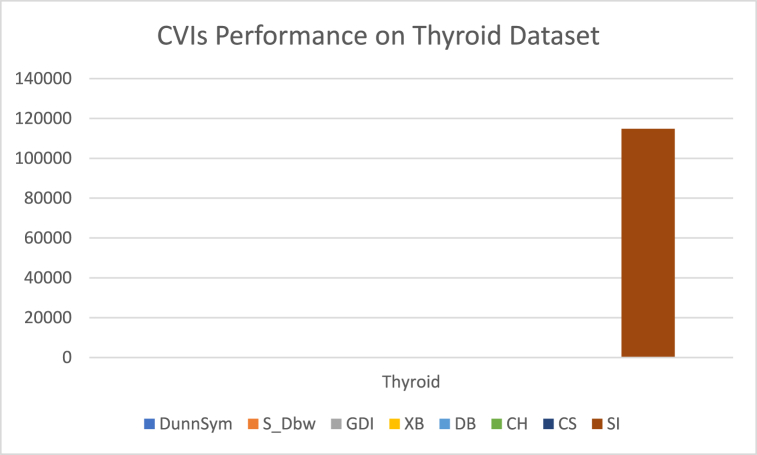
CVIs performance on Thyroid Dataset.

**Fig. 12:**
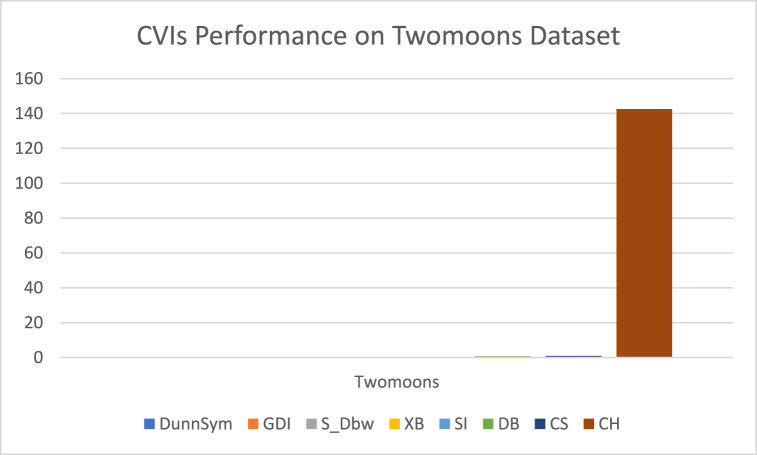
CVIs performance on Twomoons Dataset.

**Fig. 13:**
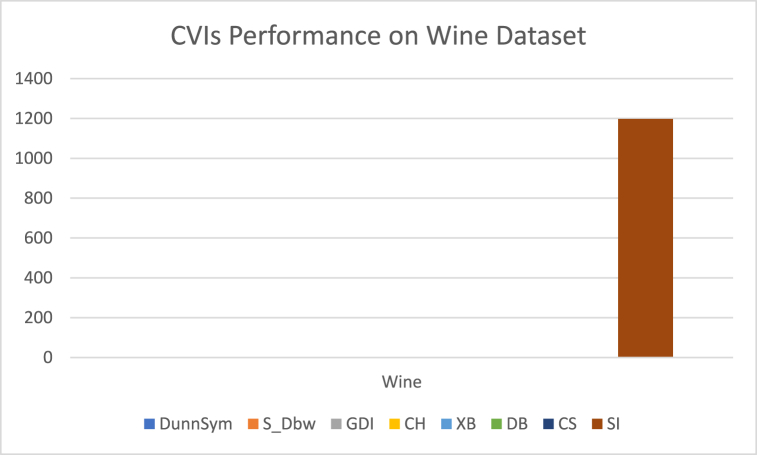
CVIs performance on Wine Dataset.

**Fig. 14:**
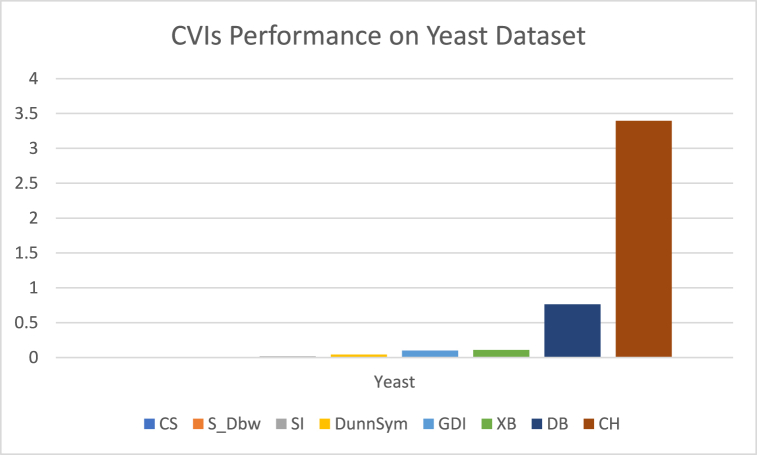
CVIs performance on Yeast Dataset.

**Fig. 15:**
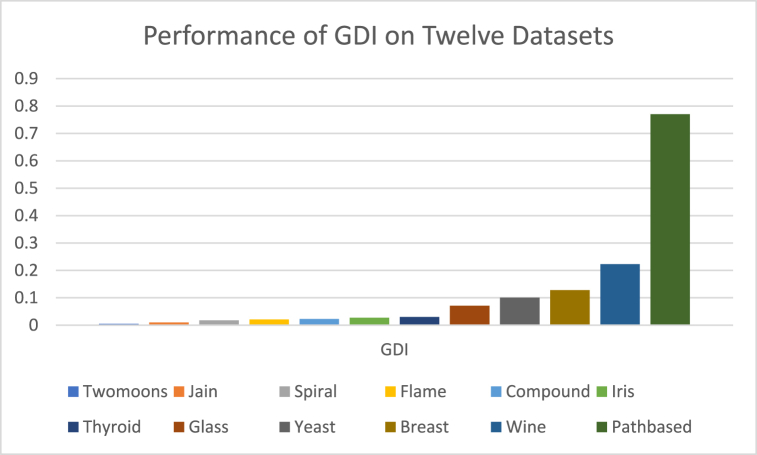
Gd index performance on 12 datasets.

**Fig. 16:**
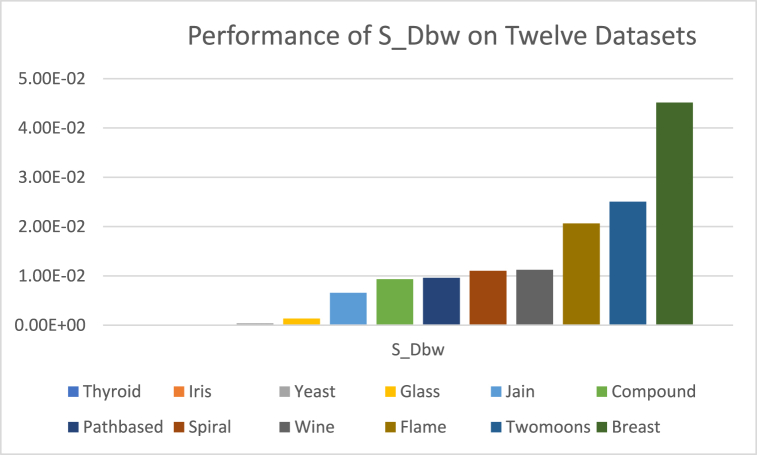
S_Dbw index performance on 12 datasets.

**Fig. 17:**
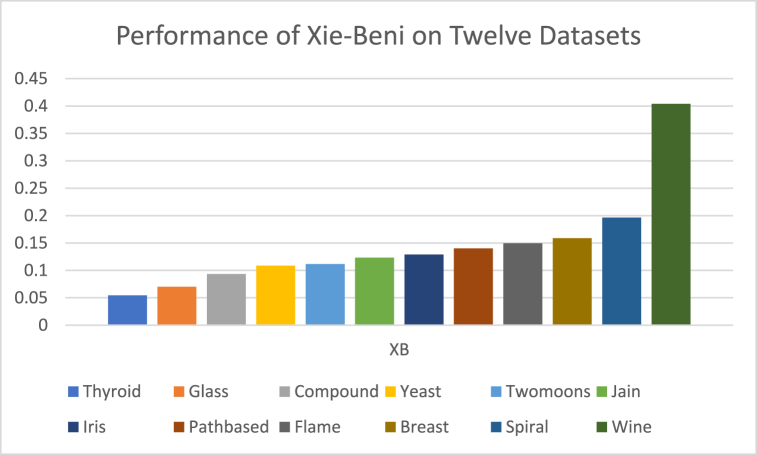
Xie-Beni index performance on 12 datasets.

**Fig. 18:**
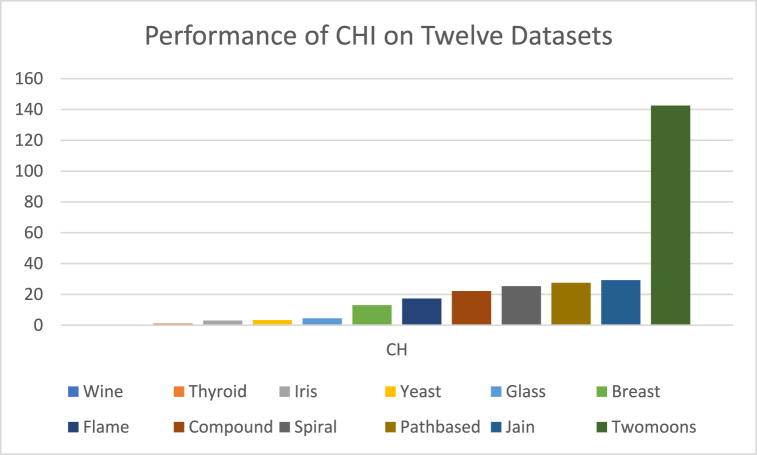
CH index performance on 12 datasets.

**Fig. 19:**
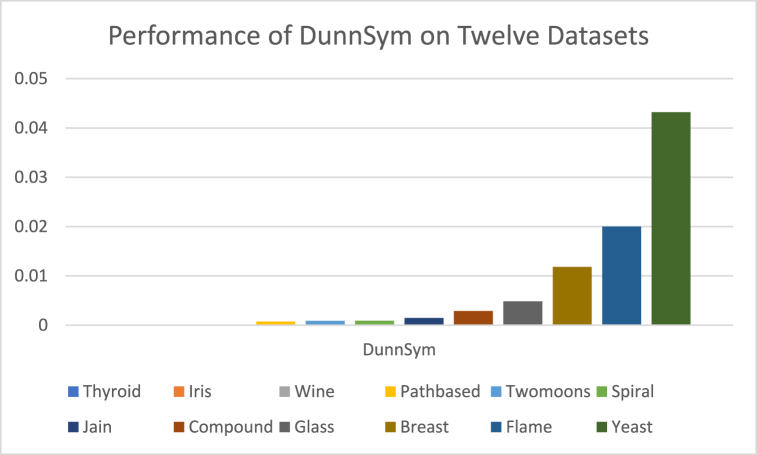
DunnSym index performance on 12 datasets.

**Fig. 20:**
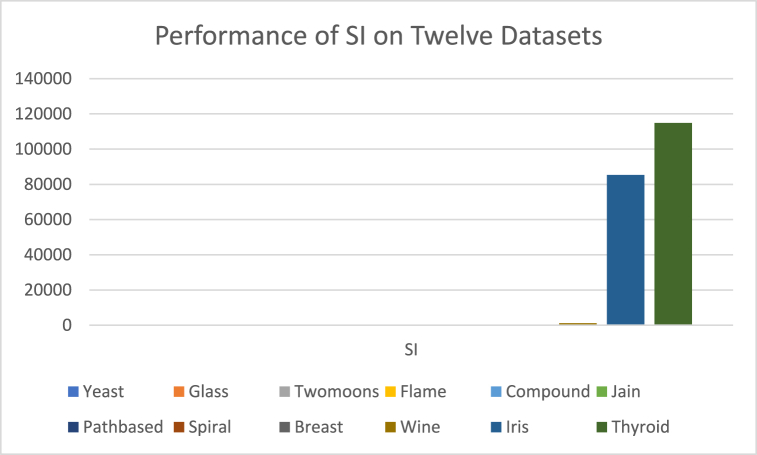
Symm index performance on 12 datasets.

**Fig. 21:**
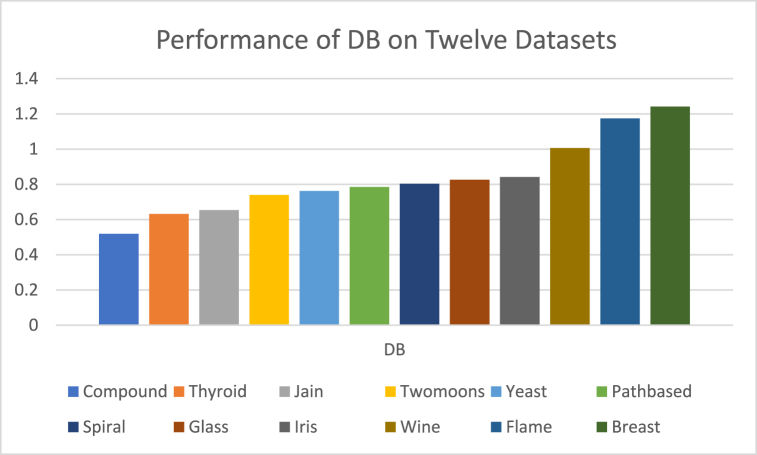
Db index performance on 12 datasets.

**Fig. 22:**
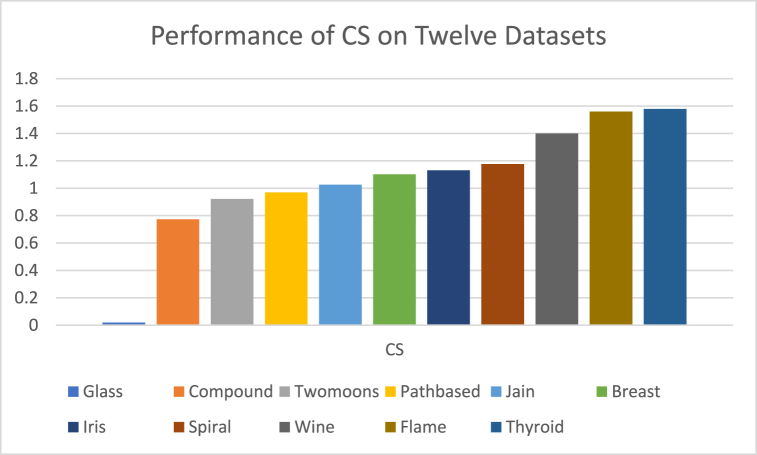
CS index performance on 12 datasets.

### 6.4 Discussion on CVIs performances on real-life datasets

The performance of the different CVIs on real-life datasets is discussed in this section. Each of the datasets has varied characteristics. For instance, the Breast, Glass, and Wine have high dimensions varying between 9 and 13 with the Glass having the highest number of clusters. However, they have a data density of less than a thousand. The Yeast dataset is characterised by high dimensionality and high density with the highest number of clusters. The performance of each of these CVIs based on these dataset characteristics will be noted in this discussion. Table 8 presents this performance based on the clustering results.

**Table 8:** Average clustering performance on real-life datasets.

	Average Clustering Performance of CVIs on Real-Life Datasets							
Dataset	GDI	S_Dbw	XB	CH	Dunn-Sym	SI	DB	CS
Breast	0.1281	0.045155	0.15874	12.9768	**0.011814**	0.700576	1.2416	1.1019
Glass	0.071372	**0.001358**	0.070395	4.52524	0.004836	0.102634	0.82578	0.02
Iris	0.027458	4.11E-05	0.128888	3.042554	**2.59E-08**	85352.2	0.84139	1.13144
Thyroid	0.030312	2.78E-08	0.054334	1.102296	**6.41E-09**	114850	0.63196	1.57881
Wine	0.222994	0.011253	0.404006	0.35299	**7.38E-06**	1197.974	1.0061	1.40034
Yeast	0.10069	**0.00038**	0.108704	3.39498	0.043199	0.018009	0.762546	0

For the average performance on high dimensional datasets, the Dunn-Sym Index and S_Dbw exhibited the best performances while SI recorded the worst performances. The GDI followed by the Xie-Beni index performed averagely well compared with DB and CS. The SI performed the worst on the Wine dataset which has the highest number of dimensions.

For the average performance on the number of clusters, the Glass and Yeast datasets have the highest number of clusters 7 and 10 respectively. The S-Dbw recorded the best performance for the two datasets followed by the Dunn-Sym. However, the SI performed better than Dunn-Sym on the Yeast datasets. The other CVIs performed averagely well.

For the average performance on the dataset density, the Yeast dataset has the highest number of objects followed by Breast with 1484 and 699 objects respectively. The S_Dbw recorded its best performance on the Yeast dataset performing better than Dunn-Sym. The SI also recorded its best performance on the Yeast dataset with a better performance compared with the Dunn-Sym. The worst performance recorded for the Dunn-Sym is on the Yeast dataset though with a better performance compared with others except S_Dbw and SI. The DB and CS recorded their worst performances on the Breast dataset. The DB recorded a poor performance on the Yeast while CS could not return any result at all.

### 6.5 Discussion on CVIs performances on synthetic datasets

The performance of the different CVIs on the synthetic datasets is discussed next in this section. Each of the synthetic datasets is generated to demonstrate different characteristics with varying degrees of complexity and overlapping. As earlier mentioned in section 6.2, the Path-based, Spiral, and Two-moons datasets represent arbitrarily shaped clusters having intertwined clusters. The Path-based dataset exhibits more complex paths compared with Spiral and Two-moons datasets. The Jain dataset is a representation of complex shapes with overlapping characteristics while the compound and flame represent datasets with non-linearly separable clusters having different shapes and densities. The performance of each of the CVIs based on these datasets with varying degrees of complexity is the point of discussion in this section. Table 9 presents the performances of the CVIs on the various synthetic datasets based on their clustering results.

**Table 9:** Average clustering performance on synthetic datasets.

	Average Clustering Performance of CVIs on Synthetic Datasets							
Dataset	GDI	S_Dbw	XB	CH	Dunn-Sym	SI	DB	CS
Compound	0.022873	0.009323	0.093588	22.1904	**0.002889**	0.59727	0.519386	0.77324
Flame	0.020888	0.020641	0.14963	17.3804	**0.01999**	0.318642	1.173924	1.55968
Jain	0.010184	0.006578	0.12338	29.1736	**0.001478**	0.607982	0.6532	1.02611
Path-based	0.77061	0.0096	0.14029	27.5826	**0.000734**	0.684518	0.784962	0.968948
Spiral	0.017882	0.011035	0.196416	25.3902	**0.000912**	0.689684	0.80329	1.17602
Two-moons	0.00483	0.025071	0.111604	142.5633	**0.000878**	0.202264	0.73901	0.922632

All the synthetic datasets are low dimensional specifically two dimensions. In terms of the number of clusters, all the synthetic datasets have just two clusters except Compound and Path-based datasets which have six and three clusters each. The report on the CVIs' performance will majorly focus on how well they can handle non-linearly separable clusters of different shapes and densities.

From the general point of view, the Dunn-Syn index recorded the best performance for all the synthetic datasets with its best performance on the Two-moons dataset and its worst performance on the Flame dataset. For the dataset with complex shapes and overlapping characteristics represented by the Jain dataset, the S_Dbw recorded its best performance. The GDI and Xie-Beni performed averagely well compared with SI, DB, and CS on this dataset.

For the datasets characterized by non-linearly separable clusters with different shapes and densities represented by the Compound and Flame datasets, the Dunn-Sym recorded the best results followed by the S_Dbw. The GDI, Xie-Beni, and SI performed averagely well in that order. The DB and CS recorded their best performance on the Compound datasets (though worse than the earlier mentioned CVIs) and recorded their worst performance on the Flame dataset.

For the datasets characterised by arbitrarily shaped clusters with overlapping clusters represented by the Path-based, Spiral, and Two-moons, the Dunn-Sym recorded the best performances with its best performance recorded for Two-moons which coincidently has the highest number of data objects. The S_Dbw performances on Path-based were better compared with Spiral and Two-moons. The performances of GDI, Xie-Beni, and SI on two moons were average better when compared with their performances on the Spiral and Path-based. The performances recorded by DB and CS are poor compared with other CVIs.

From the observed performances of the CVIs, it can be noted that the Dunn-Sym performed better than the other CVIs on the synthetic datasets and mostly so on datasets with arbitrarily shaped and overlapping clusters. The S_Dbw also recorded averagely better performances compared with GDI, Xie-Beni, and SI. The DB and CS recorded worse performances compared with the other CVIs.

To statistically validate the experimental results, a series of statistical analysis were carried out on the data. The Friedman Rank Test was carried out to detect differences among the various cluster validity indices across the multiple datasets. The Friedman Rank test [216] is a non-parametric statistical test that is mostly used when there are repeated measures such that there are the same subjects under different conditions. In this case, several cluster validity indices are tested on datasets to investigate their performances in relation to each of the datasets.

The Friedman Rank Test ranks each of the CVIS per dataset, evaluating the sum of ranks of each of the CVIs, and analyses the sums to determine if there is a statistically significant difference among them. The Friedman test statistics follows a chi-square distribution with a null hypothesis that there are no differences between the CVIs. The statistical analysis of the data obtained from the experiments produced a Friedman test statistic of 69.80 with a p-value of 1.63e-12.

The Friedman test result shows that there is a statistically significant difference among the CVIs across the datasets that were evaluated. This is indicated by the extremely small p-value which is much lower than the 0.05 null hypothesis acceptance value. Therefore, it can be concluded that at least one CVI has a significant performance difference compared with other CVIs.

**Fig. 23:**
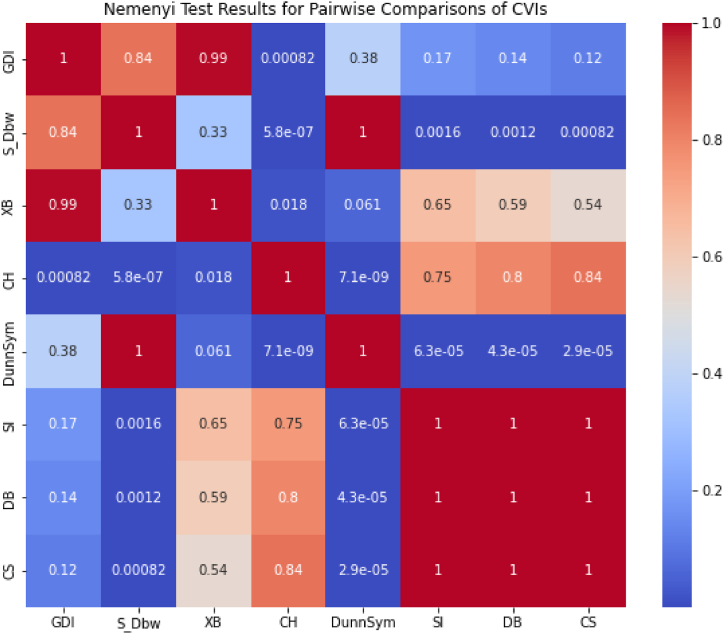
Nemenyi test results for pairwise comparisons of the CVIs.

To determine which specific CVIs differ, a post-hoc test – the Nemenyi test - was carried out to identify the pairs of CVIs that exhibit statistically significant differences. This is to show which indices outperform or underperform relative to each other. The Nemenyi test [217] is used for pairwise comparisons to determine the significant differences between the CVIs. The Nemenyi test produced a heatmap that shows the p-values for each pairwise comparison of the CVIs. The heatmap is shown in Fig. 23.

The cells with p-values <0.05 imply that there are significant differences between the CVI pairs that form the cell. This indicates that the performance of one CVI is significantly different from the other across the datasets. The red cells have a p-value ≈1 which indicates high p-values to show that there is no statistical difference between the two CVIs in that cell. This implies that performances are very similar.

The cell in the shades of blue indicates lower p-values with darker blue colours showing a p-value that is less than 0.05. This implies that there is a statistically significant difference between the two CVIs that form the cell. This indicates that the CVIs perform differently across the datasets. Because of this, the following can be observed: there is a significant difference between GDI and CS based on the approximate p-value of $8.2\times 10^{-4}$. There approximate p-value of 0.00082 is reported for GDI and DunnSym indicating a significant difference between the two CVIs.

The CH and DunnSym have an approximate p-value of $7.1\times 10^{-9}$ which shows that there is a very strong difference in the performance of the two CVIs. In the same vein, the S_Dbw performs significantly differently from the DunnSym which is indicated by the approximate p-value of $5.8\times 10^{-7}$. Moreover, the SI, DB, and CS in comparison with DunnSym show very low p-values in the range of $10^{-5}\;to\;10^{-9}$ which indicates statistically significant differences between them. However, the heatmap indicates that there are no statistically significant differences between GDI and S_Dbw as well as between SI and DB. This implies that these pairs demonstrate similar performances across the datasets. It is also shown that S-Dbw and XB, SI, and CS have high p-values which indicates that there are no significant performance differences between them.

Moreover, the confidence intervals [218] for each of the CVIs across the datasets were also estimated using bootstrap confidence intervals based on the 2.5th and 97.5th percentile of the distribution as the bounds of a 95 % confidence interval. This gives a sense of variability for each of the CVIs with respect to the different datasets. The mean and 95 % confidence interval for each of the CVIs is presented in Table 10.

**Table 10:** Mean and 95 % Confidence Interval for each CVI.

Mean and 95 % Confidence Interval for Each CVI			
	Mean	95 % CI Lower	95 % CI Upper
GDI	0.12054	0.042117	0.238925
S_Dbw	0.011754	0.005823	0.019251
XB	0.14454	0.106795	0.195972
CH	24.213927	9.903149	45.567862
DunnSym	0.007093	0.001823	0.014243
SI	16211.52378	184.725214	37392.51573
DB	0.833124	0.731929	0.943132
CS	0.967853	0.695004	1.220978

In analysing the performance of the CVIs across various datasets, the mean gives the CVI's average value across the datasets while the 95 % confidence interval indicates the range where the true mean of the CVI is expected to fall with 95 % confidence based on the variability in the data. A higher or lower mean reflects the typical measurement provided by the CVI across the datasets. A narrow confidence interval is an indicator of less variability for the CVI while a wide CI indicates greater variability.

The GDI has a relatively low mean with a relatively narrow confidence interval. It is an indicator that GDI demonstrates moderate performance consistency across the dataset. S_Dbw has a lower mean with a very narrow confidence interval compared with GDI. It indicates that S_Dbw demonstrates a high level of consistency and low variance across the datasets. The XB index has a higher mean however with a relatively narrow confidence interval. This suggests that the performance of the XB index is stable across the datasets.

The CH index has a high mean of larger values compared with other indices and a relatively wide confidence interval. This shows significant variability across the datasets. For the DunnSym index, the mean is very low with a narrow confidence interval. This suggests high consistency with low variability across the datasets. SI index has an extremely high mean with a very with confidence interval which implies a lot of variability across the datasets. This indicates that SI's performance is not stable, and it varies significantly across the datasets.

DB has a moderate mean with relatively narrow CI which indicates that its performance is consistent across the datasets. For the CS index, the mean is relatively high with a relatively wide confidence interval. This shows that CS demonstrates some variability across the datasets.

## 7 Conclusion

The Cluster Validity Index is an important aspect of clustering processes. It is employed in evaluating the quality of potential clustering solutions. Several CVIs have been proposed in the literature for clustering processes in general. CVIs are categorized into three: external, internal, and relative criteria. The internal cluster validities are employed in automatic meta-heuristic-based clustering algorithms as fitness functions for the optimization process of the clustering algorithm. Cluster validity indices are measured based on the relationship between cluster characteristics such as cluster cohesion, cluster separation, cluster symmetry, and connectedness. This study presents a comprehensive survey of internal cluster validity indexes that have been used as fitness functions in automatic meta-heuristic-based clustering algorithms. It presents the strengths and weaknesses of the various internal cluster validity indexes and the peculiar application areas. This review paper will be beneficial for both researchers and practitioners.

The findings in this review show that the Davies Bouldin index is the most used CVI for automatic meta-heuristic-based clustering algorithms followed by the CS index, Xie-Beni index, Symmetric Index, and WGS index. DB index performance however degrades when handling datasets with arbitrarily shaped clusters with varied densities. The proximity measure adopted in any cluster validity index determines the shape of the clusters that can be identified. The use of Euclidean distance identifies spherically shaped clusters while the maximum edge distance is good at discovering irregular-shaped clusters. The Cosine distance is employed mostly when priority is given to discovering the orientation between patterns rather than their magnitude.

Cluster validity indices' reliability/performance varies for the clustering method, the data structure as well as the clustering objective. Cluster overlap, experimental factors, and the presence of noise have an impact on cluster validation indices performance. Most CVIs demonstrate better results with fewer clusters. Distance among features becomes meaningless in high-dimensional datasets. In data gene expression and similar domains where the overall shape of the gene expression patterns is more important, Pearson's correlation coefficient is used in measuring the similarities in the shapes of gene expression patterns.

From the experimental results, it has been statistically validated that DunnSym has significant differences with many other indices like the GDI, S_Dbw, CH, SI, DB, and CS. Also from the statistical test, it can be concluded that GDI and S_Dbw or SI and DB exhibit similar performances. These will assist in making an informed choice of CVIs for future clustering evaluation. Based on the confidence interval for the CVIs across the dataset, it can be observed that SI and CH performances are less consistent while S_Dbw, DunnSym, and DB are more stable and reliable across the datasets. The stability and reliability demonstrated by the DunnSym and S_Dbw make them more suitable for comparative studies of clustering algorithms.

Future experimental studies can discuss the performance of these and other CVIs not mentioned here in terms of their performance with reference to different distance metrics, dimensionality, and density variation to show which CVIs perform better under specific conditions.

## CRediT authorship contribution statement

**Abiodun M. Ikotun:** Writing – review & editing, Writing – original draft, Visualization, Validation, Investigation, Data curation, Conceptualization. **Faustin Habyarimana:** Writing – review & editing, Writing – original draft, Visualization, Validation, Supervision, Software, Resources. **Absalom E. Ezugwu:** Writing – review & editing, Writing – original draft, Visualization, Validation, Supervision, Software, Resources, Methodology, Investigation, Data curation, Conceptualization.

## Ethical approval

NA.

## Availability of data and materials

All data generated or analyzed during this study are included in this article.

## Declaration of competing interest

The authors declare that they have no known competing financial interests or personal relationships that could have appeared to influence the work reported in this paper.
